# SynRoute: A Retrosynthetic Planning Software

**DOI:** 10.1021/acs.jcim.3c00491

**Published:** 2023-08-28

**Authors:** Mario Latendresse, Jeremiah P. Malerich, James Herson, Markus Krummenacker, Judy Szeto, Vi-Anh Vu, Nathan Collins, Peter B. Madrid

**Affiliations:** SRI International, 333 Ravenswood Ave, Menlo Park, California 94025, United States

## Abstract

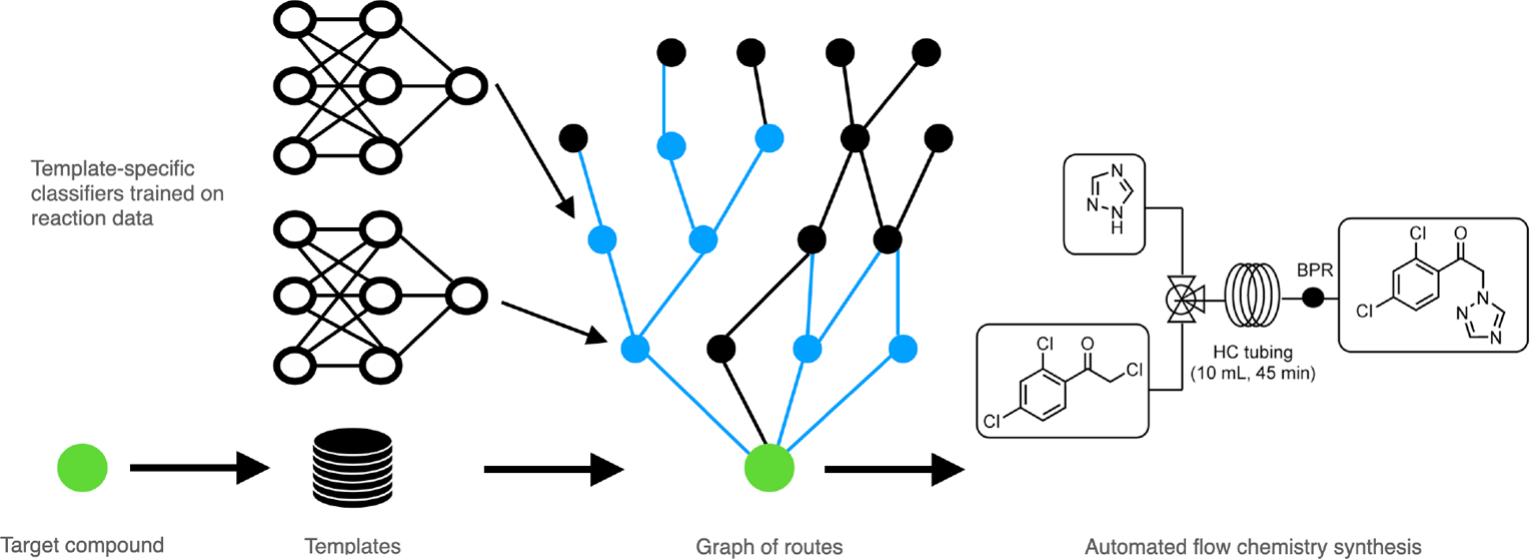

Computer-assisted synthetic planning has seen major advancements that stem from the availability of large reaction databases and artificial intelligence methodologies. SynRoute is a new retrosynthetic planning software tool that uses a relatively small number of general reaction templates, currently 263, along with a literature-based reaction database to find short, practical synthetic routes for target compounds. For each reaction template, a machine learning classifier is trained using data from the Pistachio reaction database to predict whether new computer-generated reactions based on the template are likely to work experimentally in the laboratory. This reaction generation methodology is used together with a vectorized Dijkstra-like search of top-scoring routes organized by synthetic strategies for easy browsing by a synthetic chemist. SynRoute was able to find routes for an average of 83% of compounds based on selection of random subsets of drug-like compounds from the ChEMBL database. Laboratory evaluation of 12 routes produced by SynRoute, to synthesize compounds not from the previous random subsets, demonstrated the ability to produce feasible overall synthetic strategies for all compounds evaluated.

## Introduction

The design and execution of a synthetic route has traditionally been a skill limited to highly trained experts in the field of chemical synthesis. It requires extensive searching of the chemistry literature, knowledge of many types of chemical reactions, and the ability to perform high-level strategic planning to create a viable route.

Efficient database searching tools are available in the chemical literature, but they require manual searching of individual reactions and constructing these steps into a complete synthetic plan that starts from available materials. This process requires significant training and expertise, and the large number of chemical reactions and the many ways to serialize them into routes make it difficult for chemists to find not only feasible routes but also the more efficient and economical ones.

There is a long history of computer-aided synthesis planning (CASP) software tools to help chemists synthesize molecules. The first major tool is the well-known *Logic and Heuristics Applied to Synthetic Analysis (LHASA)* from Elias Corey et al.,^1−4^ and LHASA was followed by several tools such as CAMEO^5^ and SOPHIA.^6^ An overview of the development of the CASP field can be found in Williams and Dallaston.^7^

The advent of deep learning and the availability of publicly available large reaction databases such as the ones based on patents^8^ have given a new impetus to the CASP field. There have been several new retrosynthetic planning methodologies published, and some have been commercialized into products that try to solve this problem computationally.

Some of these approaches rely primarily on computationally generated reactions from human-coded expert rules applied via retrosynthetic analysis to a target compound with heuristic route search strategies.^9−11^ Others use automatic extraction algorithms from a large corpus of reactions to identify reaction transformation templates and have applied machine learning techniques to identify applicable templates to target compound and generate new reactions that could produce a specific target.^12,13^

Another approach is to avoid the use of a fixed set of templates and instead train a deep neural network, such as a transformer, to predict the products given a set of reactants or the reverse, that is, to predict the reactants given a product. The work of Schwaller et al.^14^ predicts the products given the possible reactants. In that direction, it cannot be used to directly infer retrosynthetic reactions, but it can be used to verify the feasibility of reactions. On the other hand, the works of refs (15−17) use transformer neural networks to predict possible reactants, given a product, and can be used to create reactions retrosynthetically. However, these studies only measure the accuracy of a single step, that is, the accuracy of single reactions, and not as complete routes from purchasable compounds to target compounds. It is yet inconclusive if the performance of these template-free appproaches using deep neural networks is comparable to that using templates.

Herein, we describe a retrosynthetic planning program called *SynRoute* that utilizes computer-generated reaction strategies combined with the ability to utilize reactions from a “fixed reaction database” to develop synthetic routes from commercially available starting materials. The fixed reaction database is composed of reactions from patents (i.e., Pistachio^18^) extended with closed-loop experimental results. This combination offers a fast and practical approach for finding routes to access the synthetic compounds.

We describe the overall performance of SynRoute and how it has been specifically applied to the challenge of producing compounds on an automated flow chemistry platform called *AutoSyn* developed by our group.^19^

The combination of computational synthetic planning and automated synthesis has the potential ability to enable a much broader range of operators to become proficient at producing high-value synthetic compounds. The majority of pharmaceutical compounds is primarily synthesized using a somewhat limited set of chemical reaction types, which have been referred to as *Medicinal Chemists’ Toolbox* (MCT) transformations.^20^ The description of these MCT transformations includes a mix of relatively specific types of reactions (e.g., *Friedel–Crafts Acylation*) and more general classifications (e.g., *N-containing heterocycle formation*). Starting from this analysis, we defined a set of 263 general reaction transformations based on this set.

These common types of transformations have many examples in the chemistry literature, making them excellent candidates for the application of machine learning techniques to predict the success of computer-generated reactions during route development.

SynRoute has been designed to propose several routes ranked by a specific metric. These routes are also *diversified*; that is, they show a diversity of strategies and purchasable building blocks. The used metric combines the length of the routes with the cost of the building blocks. To that end, we have designed a route-searching algorithm that finds diversified *k* best routes.

## Results and Discussion

### Finding Diversified Optimal Routes

**Figure 1:**
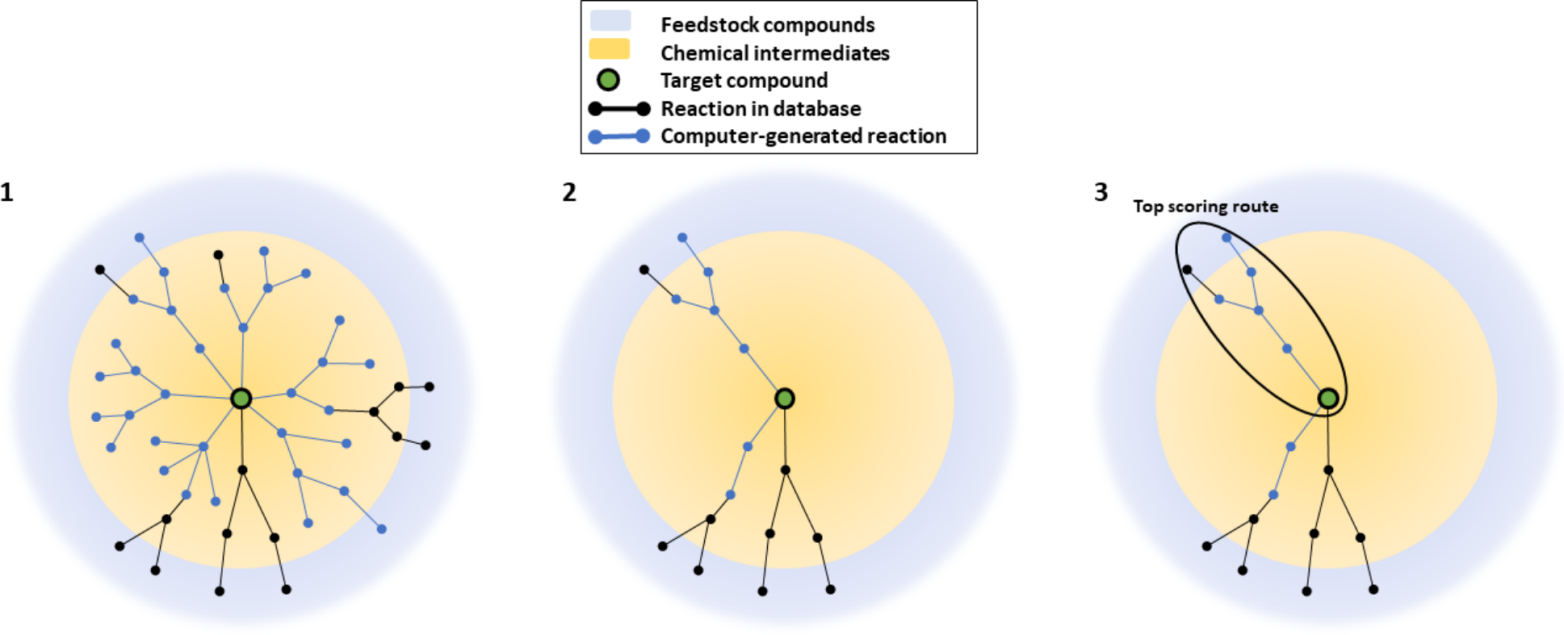
Depiction of the overall algorithm to find diversified optimal routes using computer-generated reactions combined with the fixed reaction database and building blocks database. 1) best-first retrosynthetic generation of new reactions. 2) creation of subnetwork of compounds and reactions that can connect to the target compound. 3) vectorized Dijkstra search on this subnetwork to identify the top (diversified) *k* lowest cost routes.

The searching of routes for a given target compound is done in three phases on a combination of reactions from a database (i.e., Pistachio) and computer-generated reactions (Figure 1). These phases are as follows:

1. A best-first retrosynthetic generation of new reactions from the target compound up to building blocks or to known synthesizable compounds.
2. A creation of subnetwork of compounds and reactions that can connect to the target compound.
3. A vectorized Dijkstra search on this subnetwork to identify the top (diversified) *k* lowest cost routes using a cost function based on the number of reactions in the routes and the cost of building blocks. The search is based on the well-known algorithm published by Edsger Dijkstra.^21^

We first present how transformation templates to generate new reactions were created. They are applied to generate new potential reactions to complement the reactions available in a fixed database and enable the generation of a more optimal synthetic route.

### Creation of Templates

The transformation templates to generate the reactions were created in two stages. In the first stage, we manually translated into SMARTS templates (SMIRKS according to Daylight’s acronyms^22^) the general transformations from Roughley and Jordan’s paper *Medicinal Chemists’ Toolbox* (MCT).^20^ Some of these 62 transformations required several SMARTS. All of these SMARTS can be interpreted by RDKit in the forward and reverse (i.e., retrosynthetic) direction.

In a second stage, an additional set of 201 transformations was created to handle less-popular transformations and some specific heterocycle reactions. These were either manually transcribed into SMARTS templates or the templates were first programmatically generated from reaction examples and then modified manually by combining some of them into more general SMARTS. We develop the program to automatically generate the SMARTS templates. SynRoute has a total of 463 SMARTS for 263 transformations.

This approach to creating the templates is in contrast to that described in several other published works^13,23−25^ in which all the templates are programmatically generated from a database of experimentally verified reactions. The software used to generate these templates is typically RDChiral,^26^ which is based on RDKit. In its current state, RDChiral generates specific templates, because it takes into account very specific details around the reaction center. The approach has the disadvantage of generating a large number of templates, typically in hundreds of thousands given a database of a few million reactions. The large number of templates raises several important technical issues regarding the selection of appropriate templates for doing one-step retrosynthesis.

For example, Heid et al.^24^ worked on deduplicating templates, by removing equivalent and overlapping templates, to improve the accuracy of the neural networks selecting templates for one-step retrosynthesis. This work though does not produce as general templates as the ones created in SynRoute. Fortunato et al.^27^ worked on increasing the performance of a neural network to select the appropriate templates by data augmentation, which required substantial computational resources. The main reasons to develop and implement these techniques are due to the large number of templates used.

Szymkuć et al.^28^ have studied the number of templates that can be created automatically from large sets of reactions using RDChiral. That study shows that the number of templates generated by that software from a set of *n* reactions is around . The two data sets used had 3.72 and 0.90 M reactions each, with respectively 310 K and 85K templates generated.

Segler et al.^13^ is using a Monte Carlo Tree Search (MCTS) algorithm called 3N-MCTS, because it is based on three neural nets, to find routes from the target compound to building blocks. Two sets of templates were generated from the Reaxys database. One large set of about 300000 templates is used during the expansion phases of MCTS, and a smaller set of around 17000 templates is used during rollouts. One neural network per set is used to select the best templates. A third neural net is used to determine whether the generated reactions from the templates are likely feasible in the lab. Their algorithm can return one or several synthesis plans from the target compound to building blocks in the tree constructed by the MCTS by following paths of the largest valued nodes.

This approach of using a very large set of templates, such as 300 000 or even tens of thousands, still leaves unanswered the possibility of using a much smaller set (e.g., a set of 1000 of templates based on “named reactions”) of well-curated general templates that can be used to obtain reliable routes and have the advantage of explainability in the form of named reactions. A smaller set of templates also removes the difficulty of training a very accurate neural network to select the applicable templates.

In Chematica,^11,29^ the templates (called rules in Chematica) were mostly created manually over a period of more than a decade, resulting in 75K templates.^10^ This large set of templates still needs a complex mechanism to select which rules are retrosynthetically applicable when given a compound to synthesize.

In SynRoute, this approach of using a small set of general templates and creating one classifier per template has been used. SynRoute has 463 general templates for 263 named reactions, and it is a reasonable approach as its performance is higher than AiZynthFinder.^25^ In Supporting Information Table S9, we present some of the templates implemented in SynRoute. A complete list of the name reactions implemented in SynRoute is presented in Supporting Information file ListNameReactions.txt. We believe this set of templates could be increased with a reasonable amount of work and that even higher performance could be obtained as compared to the other approaches. The advantage of our approach is the relatively small number of templates, which allows a more precise selection given a target molecule without using any neural network to select the appropriate templates.

### Retrosynthetic Expansion Algorithms

The diversity and generality of the templates are not enough to guarantee a good overall performance for finding routes to a target compound. The algorithm used to retrosynthetically expand a route from the target compound to the purchasable building blocks also plays a major role in overall performance.

Starting from the target compound, a *retrosynthetic expansion algorithm* uses applicable templates to generate one or two reactants. This is a one-step expansion. These reactants and the target compound form a generated reaction. This process is iteratively applied on the reactants if these reactants are not purchasable building blocks or are known to be synthesizable from the fixed set of reactions of the database. The result is an acyclic graph of the generated reactions. A route is obtained if at least one path exists from the purchasable building blocks to the target compound. The selection of nonexpanded reactants on which to apply templates forms the core of a retrosynthetic expansion algorithm. We studied the performance of four different algorithms to do that selection: breadth-first, depth-first, best-first compound, and best-first reaction. A general presentation of these algorithms is described in a book by Russel and Norvig.^30^

We have compared the performance of SynRoute to the published results of Segler et al.^13^ and Genheden et al.^25^ for AiZynthFinder (Table 1). The comparison is complicated by the use of different benchmarks, the computational time limit, and the purchasable compound databases described in these papers. We used several benchmarks to show the stability of the SynRoute overall approach. Instead of using diverse computational time limits, we have used several different maximum numbers of generated reactions, which can easily be reproduced by other researchers, because a time limit is too dependent on the underlying hardware used. With the comparison done, for which all the results are shown in Table S1, it shows that the overall approach of SynRoute is satisfactory and even superior to previously published results across three varied parameters: the expansion algorithm, the benchmark, and the maximum number of generated reactions. The database of purchasable compounds used is not varied. We used the eMolecules database of 2022 (Q4), and only “Building Block” compounds classified as tier 1 (shipped within 2–5 days) or tier 2 (shipped within 2–10 business days) were included.

**Table 1:** Six Benchmarks Used to Test the Performance of SynRoute^a^

**Benchmark**	**OverallavgSascore**	**Top 30avgSascore**	**SMILESsolved bySynRoute**
AiZynth	2.80	3.66	86
Bench 1	3.12	4.31	77
Bench 2	3.08	4.46	87
Bench 3	2.99	4.25	78
Bench 4	3.02	4.08	79
Bench 5	2.85	3.80	90
Avg	2.97	4.09	82.8

a Each benchmark had 100 SMILES selected randomly from the ChEMBL database. The number of SMILES solved by SynRoute shown on the right most column is from the best-first reaction expansion algorithm, used in the current implementation of SynRoute, using a maximum of 50K generated reactions (see Supporting Information Table S1 for the detailed timing of each benchmark).

In the case of Segler et al.,^13^ the benchmark is a set of 497 randomly selected compounds extracted from clustered compounds generated from 12.1 M reactions of the Reaxys database, which is not publicly available. The benchmark does not include compounds that were not seen during extraction of the templates. Unfortunately, this approach does not test the generalization capability of the templates because the templates were extracted from the same set; therefore, any synthesizable compounds in the Reaxys database will likely be synthesizable by the templates.

Genheden et al.^25^ have made available the 100-SMILES benchmark extracted from the ChEMBL database and used for AiZynthFinder, but the database used to extract templates and train neural networks is not Reaxys; it is from a smaller database created from the USPTO patents. The technique used to extract the templates is similar to Segler et al.^13^ The lack of generality of the templates is confirmed by the AiZynthFinder paper,^25^ because their benchmark of compounds was extracted from a different database (i.e., ChEMBL) than the database used to extract templates (i.e., USPTO), and the percentage of SMILES solved (55–65%) is much lower than the percentage reported by Segler et al.^13^ (95%).

We have used six benchmarks to test the performance of SynRoute: the publicly available AiZynthFinder benchmark^25^ and five benchmarks we created by randomly selecting, for each benchmark, 100 compounds from the ChEMBL database. We decided to use five additional benchmarks of 100-random compounds instead of a single benchmark of 500-random compounds to more clearly compare our results with the results of the AiZynthFinder benchmark of 100-random compounds. Indeed, the publicly available performances of AiZynthFinder and ASKCOS on the AiZynthFinder benchmark give us a point of comparison. We think the additional five benchmarks are needed to better confirm the performance of synthesis planning software. The five 100-SMILES benchmark files are described in the Supporting Information. Table 1 shows statistics on these benchmarks and the performance of SynRoute when using the best-first reaction expansion algorithm. Note that the given performances vary based on the expansion algorithm used to generate new reactions leading to routes from building blocks, and some of these algorithms do not exhaustively expand all possible routes but make an informed decision to reach purchasable compounds.

The synthesis accessibility scores (Sascores) are an estimation of the difficulty to synthesize a compound devised by Ertl and Schuffenhauer^31^ and slightly modified by Ertl and Landrum.^32^ For each benchmark, we present the average Sascores for its 100 compounds and its highest 30 Sascores, the number of target compounds that exist in the Pistachio database, and the number of compounds for which SynRoute could find at least one route.

The performance of SynRoute on the AiZynthFinder benchmark is 85%, substantially higher than the published numbers 55–65% for the AiZynthFinder tool and 62–72% for the ASKCOS tool,^25^ for which the variations 55–65% and 62–72% depend on the purchasable databases used. This comparison primarily depends on the algorithm for the retrosynthesis expansion of generated reactions and the set of templates used. This comparison also depends on the data used to train the classifiers to evaluate the probability of success of generated reactions by the templates, but to a lesser extent because their main impact is the ordering of routes found based on these evaluations. Moreover, this comparison does not take into account the feasability of routes found because such a parameter has not been published for AiZynthFinder and ASKCOS.

### Graphical User Interface (GUI)

**Figure 2:**
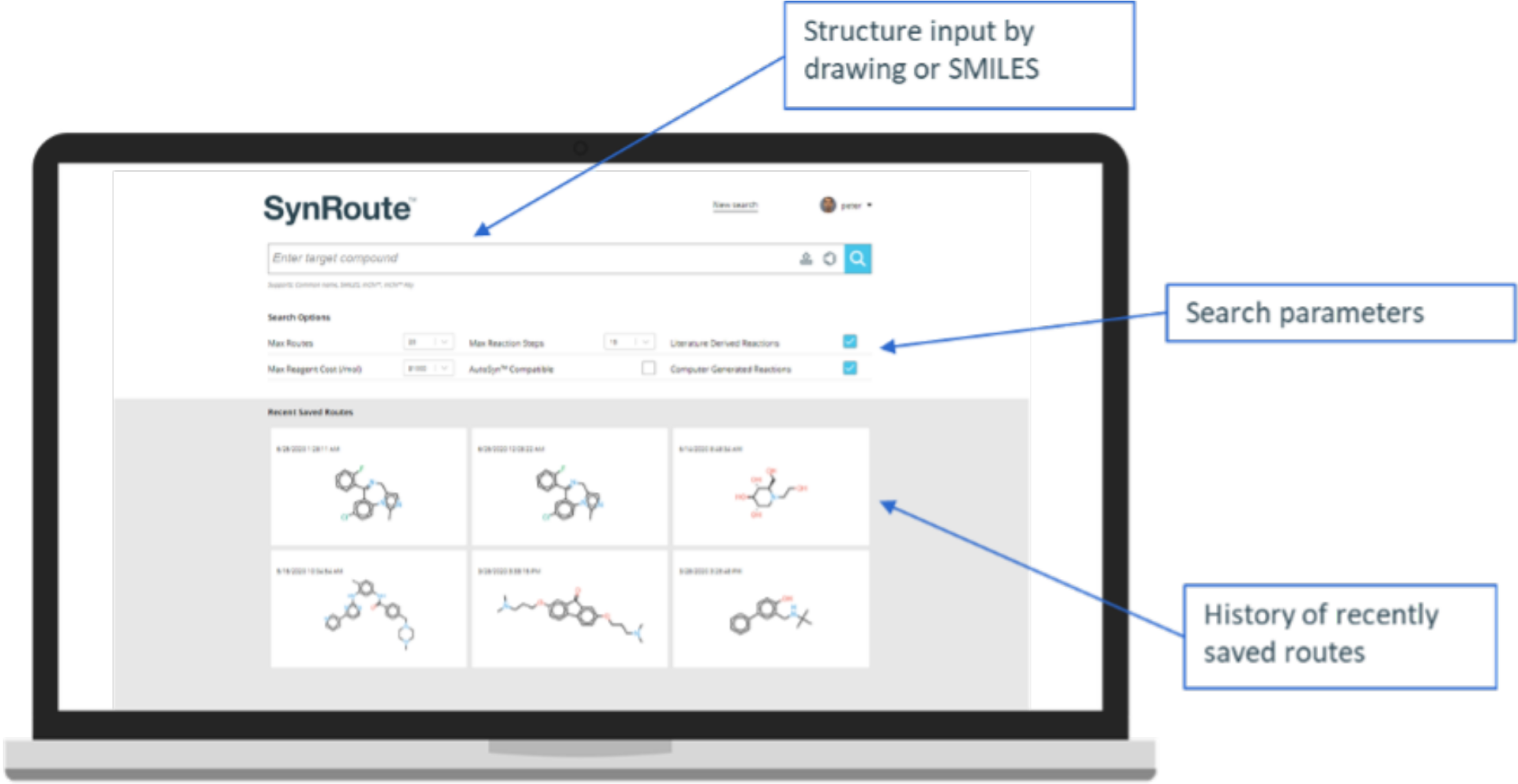
Graphical User Interface (GUI) of SynRoute for executing a route search. The compound to synthesize can be drawn using the embedded JSME editor^33^ or by entering directly its SMILES representation.

SynRoute is operated by chemists as a web application using an intuitive Graphical User Interface (GUI). The initial web page allows the chemist to specify a target compound either by using a SMILES, a compound name, or an InChI or by drawing a molecular structure using JSME^33^ and initiating the search for routes. Some parameters to control the search are also provided (Figure 2). Once routes are found, they are grouped and summarized on the *Strategies Page*. A strategy is characterized by the reaction directly producing the target compound (see Supporting Information Figure S10 for an example of a Strategies Page). The current response time for a search for multiple routes is between 15 s and two min, depending on the given target compound to synthesize, and most often less than a minute. The current implementation of SynRoute uses modest computational resources: a single CPU core (no GPUs).

Typically, several routes are grouped under each strategy. The chemist can select one of the strategies to see all the routes under that strategy (see Supporting Information Figure S11 for an example of a Strategy Page) and then select one route that appears promising for a more in-depth analysis and visual presentation. A complete route is presented as one or several linear segments similar to published synthesis routes in chemical journals (Supporting Information Figure S12 for an example of a route). At that level, any compound shown as a structure can be clicked to display more data on the left panel, such as SMILES, InChI, weight, purchasable price, if applicable, and more (see Supporting Information Figure S13 for an example on the left panel). Similarly, any reaction shown as an arrow can be clicked to display the various conditions (i.e., times, temperatures, solvents, reagents, catalysts) of the reaction, the source of the literature for a fixed reaction, and more. The generated reactions from templates are clearly identified as such using a blue color with the name of the transformations used.

If desired, routes can be iteratively refined and modified to a chemist’s satisfaction by constraining new searches with the *keep* and *avoid* functionality on reactions and compounds (see Supporting Information Figure S14 for an example of the *keep* and *avoid* buttons). A chemist can select a set of compounds and reactions to keep or avoid and initiate a new search. The routes found will necessarily have all of the compounds and reactions that were selected to keep and have none of the compounds and reactions that were selected to avoid.

Routes can be saved, printed, and shared with colleagues via email. SynRoute automatically bundles the relevant information to be sent to selected email addresses chosen by the chemist. Saved routes can also be used to provide reaction condition data for building chemistry automation protocols. The reactions, compounds, and routes can individually be flagged as dubious, so that a curator can be alerted of the issues.

### Laboratory Evaluation of Routes

We evaluated the ability to translate routes directly from SynRoute to our synthesis automation hardware, called AutoSyn, developed at SRI.^19^ Our goal is to fully automate this translation process, but a number of challenges still remain that require chemists to manually modify routes for execution on an automated chemistry platform. AutoSyn is a continuous flow chemistry platform; therefore, these changes often involve modifying reagents for greater solubility and methods for accelerating reaction times. Also, limitations of literature data still require reaction scouting and optimization before execution in the production mode. As a demonstration of the process of adapting a SynRoute route onto AutoSyn, we begin with a route for the antifungal medication itraconazole, which was initially described by Szeto et al.,^34^ followed by the potent anticancer drug bortezomib, which was initially described by Vu et al.^35^

#### Itraconazole

**Figure 3:**
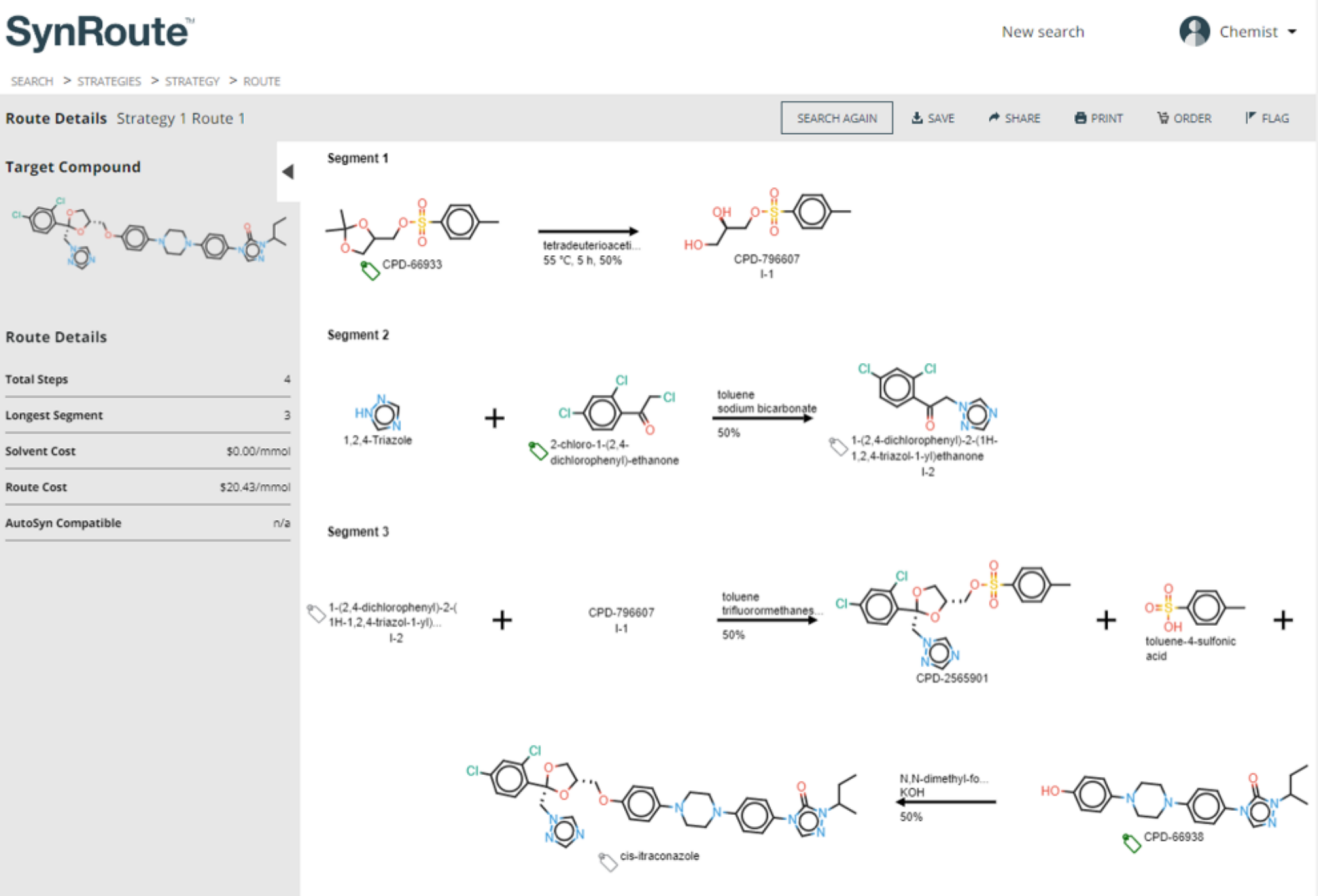
SynRoute’s top-scoring strategy for the preparation of itraconazole.

**Figure 4:**
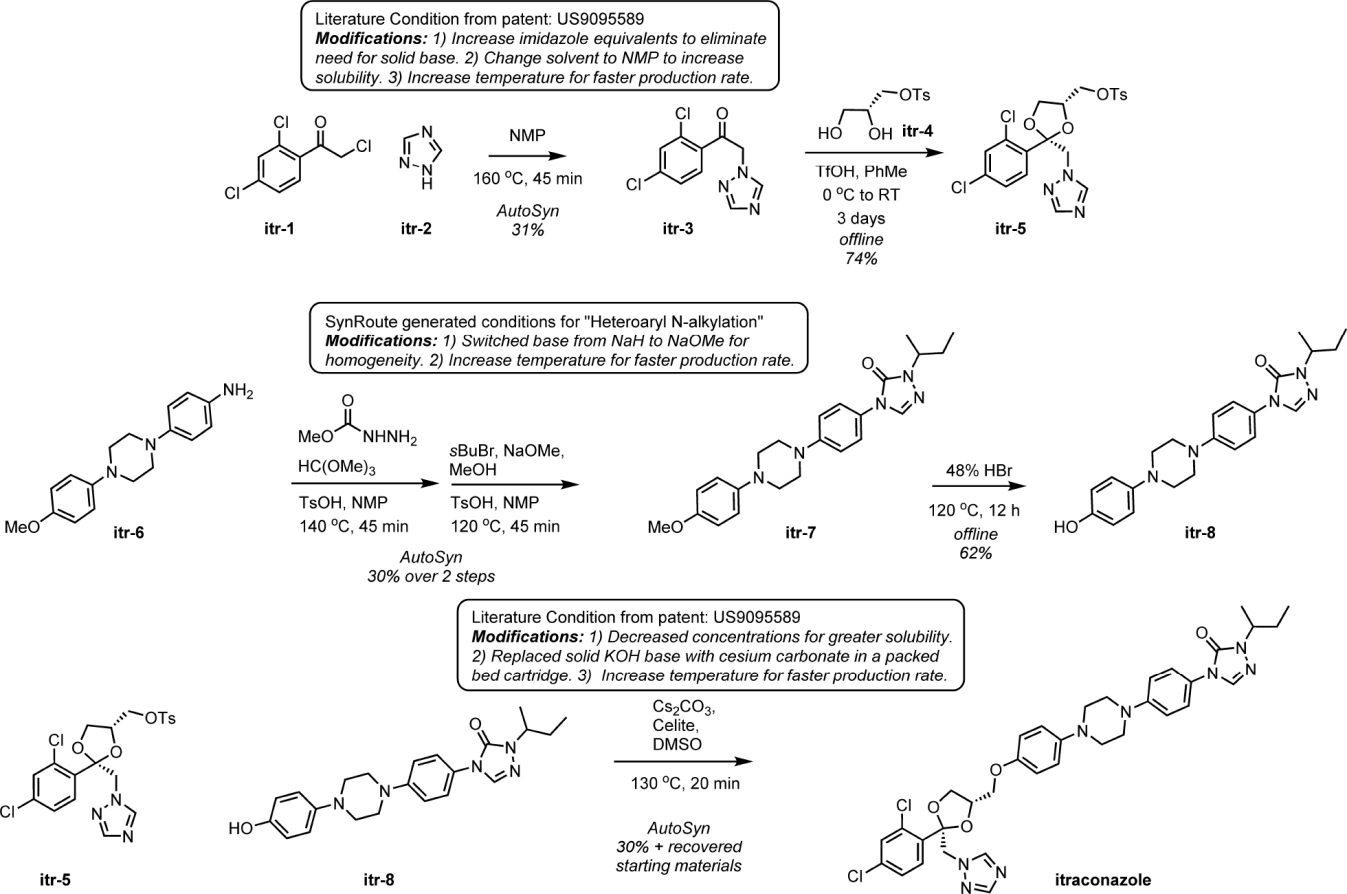
Itraconazole scheme was performed on the AutoSyn platform. For reactions that were modified for adaption to the AutoSyn flow chemistry platform, the source of the reference conditions is given along with a rationale for why they were modified.

The top-scoring route for itraconazole involved three different linear segments ending in a two-step coupling sequence to connect the halves of the final compound (Figure 3). Itraconazole is a moderately complex small-molecule drug structure with multiple chiral centers that was approved for use in 1992, so there is a lot of prior data on synthesizing this exact compound. Unsurprisingly, the top route is therefore comprised of only fixed reactions present in the Pistachio database. Performing the search without enabling fixed reactions returned several strategically similar routes, most of which were significantly shorter by using commercially available advanced intermediates (Supporting Information, Figure S1). In the top-scoring route, CPD-66938, is also an advanced intermediate that SynRoute suggests purchasing, but in practice, we chose to synthesize this intermediate to develop a lower-cost production process. A SynRoute search performed on this intermediate (CPD-66938) returned several routes with triazole alkylation reactions, but no routes that involved the triazole formation reaction from an aniline precursor. Heterocycle formation reactions, such as this triazole formation, tend to have few examples in reaction databases, like Pistachio, and, therefore, can be a limitation for machine learning-based (ML-based) synthetic planning approaches. The rest of the steps for the top-scoring itraconazole route were all performed on our AutoSyn automated flow chemistry platform with manual modifications to the reaction conditions to make them more suitable for continuous flow chemistry (Figure 4).

The synthesis of itraconazole was performed by using the route from SynRoute with a late-stage coupling of two elaborated precursors shown in Figure 4. Toward the first precursor, itr-5, itr-2 was alkylated with itr-1 using AutoSyn to give ketone itr-3. SynRoute suggested conditions of sodium bicarbonate in toluene that were modified to heating in NMP and allowing the triazine to act as a base for capturing the generated HCl. The ketalization of itr-3 with desymmetrized glycerol itr-4 must be performed at low temperature to achieve reasonable diastereoselectivity (5:1). The long reaction time (3 days) is not well suited for AutoSyn, and consequently, the preparation of itr-5 was performed offline according to SynRoute conditions. Toward the second precursor itr-8, itr-6 was pumped into AutoSyn and treated with methyl carbazate, trimethyl orthoformate, and catalytic toluenesulfonic acid to build up the 1,2,4-triazol-3-one scaffold. Introduction of *sec*-butyl bromide and sodium methoxide affected the *N*-alkylation and gave itr-7, which was performed in a 2-step telescoped process on AutoSyn. Deprotection of the phenol group of itr-7 with HBr afforded itr-8. The corrosive nature of HBr required this reaction offline. To complete the synthesis, itr-5 and itr-8 were dissolved in DMSO and passed through a heated packed bed of cesium carbonate on AutoSyn to give itraconazole. SynRoute suggested conditions with potassium hydroxide in DMF, requiring long reaction times, so they were modified to conditions that used DMSO as a solvent and cesium carbonate mixed with Celite loaded into a packed bed cartridge as a base. For more information about each step of the synthesis of intraconazole, see the Supporting Information.

This example of going from a SynRoute route to an experimental process for itraconazole demonstrates both the utility of a synthetic planning tool, such as SynRoute, for rapidly producing viable synthetic strategies and the challenges of adapting these strategies to the laboratory. In this case, many of the challenges were associated with adapting batch chemistry procedures into flow chemistry methods, but there are also important experimental considerations, such as reagent compatibilities and effects of temperature on diastereoselectivity that are not handled by most synthetic planning programs, including SynRoute.

#### Bortezomib

**Figure 5:**
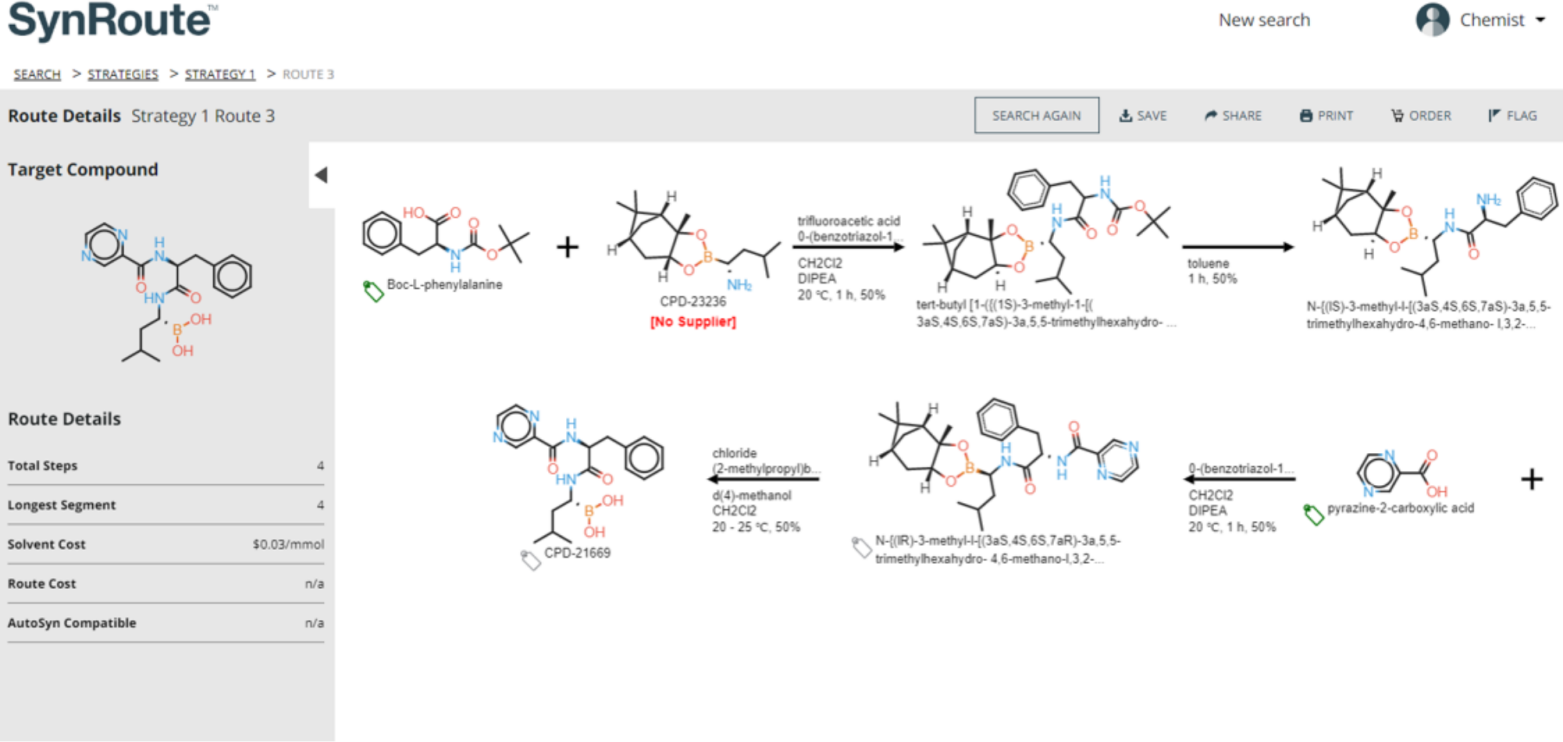
SynRoute’s top-scoring strategy for the preparation of bortezomib.

**Figure 6:**
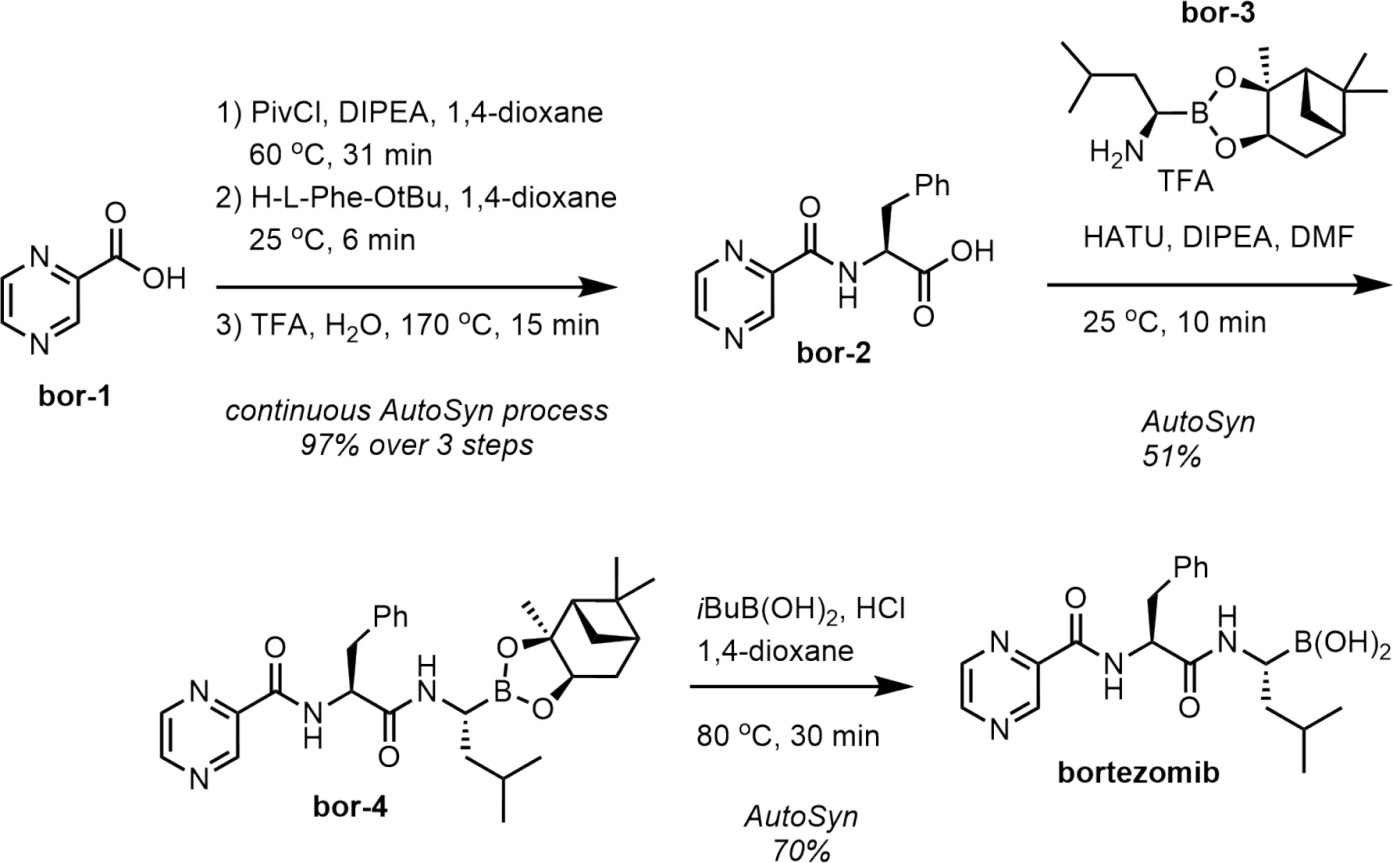
Bortezomib scheme on the AutoSyn platform.

SynRoute often provides an effective overall strategy for preparation of a target compound but may overlook the requirements around protecting groups. Such is the case for bortezomib and one of the top scoring routes found in SynRoute (Figure 5). In this example, SynRoute returned a four-step route that required a chiral boronic ester building block material without any supplier listed in our feedstock database. Routes containing building blocks without a known supplier are labeled “partial routes” and heavily penalized in the route scoring function. In this particular case, the chiral boronic ester building block was actually available from suppliers not fully covered in our SynRoute building block database. In practice, bortezomib was synthesized on AutoSyn by the route shown in Figure 6. Carboxylic acid bor-1 was activated as the pivaloyl chloride and then reacted with phenylalanine *tert*-butyl ester. The *tert*-butyl group was deprotected with TFA, and bor-2 was isolated and purified from the effluent for AutoSyn. In a second AutoSyn process, carboxylic acid bor-2 was coupled with chiral amine bor-3 using HATU. Compound bor-4 was purified offline and resubmitted to AutoSyn to deprotect the boronic ester functional group and complete the synthesis of bortezomib. For more information about each step of the synthesis of bortezomib, see the Supporting Information.

These examples illustrate the types of changes often required to adapt routes from SynRoute to a chemistry automation platform. A description of the modifications made to the SynRoute routes for a broader set of compounds is shown in Table 2. The detailed synthetic processes for these compounds are described in a prior publication.^19^ In these examples, the changes to the routes were primarily strategic decisions about whether to make or buy intermediates and minor changes to bond formation strategies. This demonstrates that we are nearing a point where computer-generated synthetic plans can be directly executed on automated synthesis platforms, but currently, an expert synthetic chemist is still required to make modifications to the detailed experimental plan.

**Table 2:** Modifications Made to the Routes Found by SynRoute Were for Successful Implementation on the AutoSyn Flow Chemistry Platform^a^

**Target**	**Chemist modifications to route for execution**
Bortezomib	Buy advanced intermediate, protecting group differences
Bupivacaine	Use acid chloride instead of acid
Diazepam	Exact route
Fluconazole	Synthesize suggested intermediate (1 step)
Hydroxychloroquine	Buy advanced intermediate
Ibuprofen	Synthesized advanced intermediate rather than purchase
2-(4-*iso*butylphenyl)propanenitrile	Buy advanced intermediate
Imatinib	Use acid chloride instead of acid (“advanced intermediate”)
Itraconazole	Buy advanced intermediate, developed new chemistry based on results observed in following SynRoute
Nevirapine	Use acid chloride instead of acid (“advanced intermediate”)
Quinapril	Buy advanced intermediates
Warfarin	Exact route

a All searches were performed enabling a combination of template-generated reactions and fixed reactions in the Pistachio database.

## Methods

### Training of Template Classifiers

After a template is applied as a one-step retrosynthesis, the feasibility score of the generated reaction, which is a value in the interval from 0 to 1, is estimated by a classifier. If the score is below 0.2, the generated reaction is rejected; otherwise, it is kept in the expanded network of reactions. The score is directly used to assign a yield to the generated reaction. This yield is used to evaluate the cost of producing the product, which is the sum of the costs of the reactants divided by the yield. This is also the same formula used to evaluate the cost of producing a product for any fixed reaction from the database of reactions. The overall costs of routes are used to order them when shown to a user.

As previously discussed, we do not use a classifier to select templates to apply, a common technique used by other retrosynthetic software tools, but only to estimate the feasibility of reactions. We generated one classifier per template if enough positive examples are available. In the following, we present how the positive and negative examples were extracted from the Pistachio database.

We programmatically selected a set of 2.77 M (2,774,796) single-step reactions, with at most two reactants and one product, from the Pistachio database (last quarter of 2021) as potential training examples for the template classifiers. Among the 2.77 M reactions, 2.28 M (2,285,860) were classified under a named reaction by Pistachio. Among them, 988 named reactions have at least 50 reactions and 810 at least 100 reactions.

From that set, positive examples for each template were identified as reactions with a yield equal or greater than 10%, or no yield was reported, matching the reactant(s) and product of the template.

Negative examples for one template were identified as reactions with a yield of less than 10%, with a complete match of the template, or as reactions matching the reactant(s) but not the product of the template, whatever their yield. Most negative examples are derived in the latter way.

It is possible that some selected negative examples are wrong, because the reported conditions of the reaction may not have produced the expected compound of a template. For example, if a different solvent or reagent had been used, the reactant(s) of that reaction would have produced the expected product for that template, contrary to the assumption that it was not produced, and therefore, it was a positive example.

The 263 SynRoute transformations, implemented using 463 templates as SMARTS, partially or entirely cover 594 Pistachio reaction class names, with 1,088,748 reactions. These reactions are positive examples for training classifiers for the templates. A relatively small number of reactions, that is, 697, were shared among six templates, which shows that the 463 SynRoute templates are largely independent. Among the 2.77 M reactions, 1.52 M of them were used as negative examples for the templates. They formed 3.62 M (3,620,684) negative examples for the entire set of templates because templates may share the same negative example reactions. Among the 463 templates, 37 templates had no positive examples, but most importantly, 70 templates had fewer than 20 positive examples, which was the threshold needed for positive examples to create a classifier for any template. That resulted in the creation of 393 classifiers.

For training the neural models, positive and negative examples, that is, reactions are encoded by representing their reactant(s) and product using 2048 bits of ECFP4 per reactant and product. There is always a maximum of three molecules because the templates have only one or two reactants.

The neural model used for all of the classifiers, one per template, is a multilayer perceptron of one hidden layer of size 10. That layer size was selected after a hyperparameter search among sizes of one or two hidden layers, varying from 10 to 50 by increments of 10 for one or two layers. We found that a model of one layer of size 10 was sufficient to obtain good performance. The sets of examples, positive and negative, are divided into three subsets, that is, 80% for training, 10% for testing, and 10% for evaluating the classifiers accuracies. The average accuracy of the 393 classifiers is 89.85%.

### Evaluation of Retrosynthetic Expansion Algorithms

In this section, we present the detailed evaluation of the four expansion algorithms mentioned in the section *Retrosynthetic expansion algorithms*, that is, the breadth-first, depth-first, best-first compound, and best-first reaction expansion algorithms.

The breadth-first algorithm is uninformed: it does a one-step expansion of all of the leaf reactants before further expanding any other reactant. It is the most systematic expansion that has the advantage of finding the shortest routes, but its major disadvantage is that, in the case of long routes, it takes hours of computational time, which is not practical.

The depth-first algorithm is also uninformed: it tries to further expand the latest reactants going as deep as possible until a maximum depth is reached or until the reactant is known to be synthesizable or can be purchased. It has the advantage of finding deep routes early but the disadvantage of often missing obvious short routes in a few seconds or minutes of computational time.

A best-first algorithm is informed: based on an estimated *complexity* score on each reactant, it selects to do a one-step expansion on a leaf reactant that is likely the easiest to synthesize. On the other hand, the depth in the expansion tree is taken into account: the deeper a reactant is, the less likely it is selected. This last criteria is used to prefer reactants that are closer to the target compound for reactant complexities that are equal or near equal, resulting in shorter routes. We have designed two different scoring functions; that is, we have two different best-first algorithms: best-first compound and best-first reaction.

For the best-first compound, the minimum scored reactant is selected for a one-step expansion, whereas for the best-first reaction, the reactants from the minimum scored reaction are one-step expanded. The score of a reactant is based on its number of heavy atoms multiplied by a factor based on the distance, or depth, from the target compound. The score of a reaction is the maximum score of its reactants multiplied by the same factor. The factor is *d*^α^ where *d* is the depth and α is a small constant. We have experimentally evaluated several values for α and found that 1.1 was producing the highest number of SMILES with routes for the AiZynthFinder benchmark when using a maximum of 25000 generated reactions. The values 0.9 and 1.2 give slightly lower results.

As shown in Supporting Information Table S1 for the performance of four expansion algorithms, the best-first reaction algorithm is the best performer across all benchmarks and various maximum numbers of generated reactions. In particular, for the AiZynthFinder benchmark, routes were found for 85% of the compounds, when using a maximum of 50K generated reactions. Increasing the maximum number of reactions to 100 K solved one more compound, and at 300 K, another one is solved. To get more compounds solved would likely require more transformations.

A deeper analysis of the set of SMILES in the AiZynthFinder benchmark reveals that for five SMILES, fewer than 5000 reactions could be generated for them. That is, at some point, for each of these five SMILES, no more one-step expansion could be done for any leaf reactants in the expansion tree to reach purchasable building blocks or known synthesizable compounds. In other words, increasing the maximum number of generated reactions will not help in finding routes for them. Only new template transformations or building blocks would help to solve these SMILES.

### Extracting Optimal Routes

Once reactions and compounds are generated by the templates to generate a graph, a two-phase, optimal route, graph-searching algorithm is applied to find multiple low-cost, typically short, and high-yielding feasible routes. In the first phase, all of the reachable compounds and reactions from the target compound, to a given maximum depth, are identified, including the purchasable compounds. That includes the generated compounds and reactions connected to the reactions and compounds from the database. In the second phase, a vectorized Dijkstra algorithm that can find multiple diversified routes from the graph and is ordered by an evaluation function is applied. The evaluation function is based on the cost of its purchasable compounds, the number of its reactions, and their yields.

The single value Dijkstra algorithm is a well-known minimum cost route searching algorithm in a directed graph published by Edsger Dijkstra.^21^ In our case, the nodes of the graph are the identified compounds of the first phase, including the target compound and the identified purchasable compounds, and the arcs are the reactions. In the original version of the Dijkstra algorithm, a node may receive only a single value (i.e., the cost). In the vectorized version used, a node, that is, a compound, may receive more than one value. In particular, the target compound may receive *k* values where *k* is the maximum number of routes requested by the chemist. All other compounds may be assigned *k* values or fewer values. In particular, the purchasable compounds for which there are no reactions producing them receive a single value, namely, their real cost from a specific vendor. These costs are obtained from the eMolecules database used by SynRoute, although this database can easily be extended from other databases if need be.

At each step of the algorithm, the minimum cost reaction is selected to set its product as the next minimum compound value in the graph. The minimum cost reaction can be efficiently identified using a heap, that is, a priority queue. Initially, the heap contains the reactions for which all their reactants are purchasable, that is, all reactions for which a cost can be immediately assigned without any search. The cost of such reactions is the sum of the cost of the reactants divided by their yield. The yield of any fixed reaction from the database is used, if known; otherwise, a default yield of 50% is used. This default evaluation is used to favor fixed reactions for which a yield has been published. The yield of a generated reaction is the probability of the feasibility of the reaction determined by the neural network of the template used to generate that reaction with a maximum yield of 70%. This hard cutoff has been set to favor a mixed use of generated reactions over fixed reactions, from the database.

Typically, the search for diversified optimal routes is substantially faster than the generation of new reactions from transformation templates.

## Conclusions

SynRoute is an effective route-designing tool that rapidly produces sensible routes for a wide breadth of compounds by using a relatively small set of 263 general reaction transformations. The routes are biased toward well-studied types of chemistry in which sufficient data are available for using machine learning methods to predict the feasibility of each computer-generated reaction. The use of SynRoute in our laboratory has shown that viable routes are usually found for moderate complexity drug-like compounds, though adapting the routes to the laboratory can require changes to reaction steps, particularly when adapting them to be performed on a continuous flow chemistry automation platform, like AutoSyn. SynRoute employs an intuitive and easy to use GUI that allows chemists to rapidly organize and browse through routes for selecting a route to be performed in the laboratory.

The true validation of any synthetic planning tool is whether routes can be performed in the lab. This is often not feasible on a statistically meaningful scale; therefore, alternatively, we introduced five new benchmarks, each composed of 100-random compounds taken from the ChEMBL database, to measure the performance of chemical synthesis planning software. Using these benchmarks, we demonstrated the performance of four multistep expansion algorithms. We have shown that a best-first multistep expansion algorithm based on the selection of reactions instead of compounds showed substantially better performance than three other expansion algorithms across all benchmarks, including the AiZynthFinder results.

The laboratory demonstrations in this work focused on well-studied drug compounds and developing continuous flow chemistry production processes. These demonstrations most closely mimic the type of planning performed during lead optimization stage drug discovery programs, where syntheses need to be scaled up and the overall process efficiencies are evaluated more rigorsly. During earlier stage drug discovery programs, SynRoute is sufficiently fast (30–60 s per compound) to score hundreds to thousands of compounds for synthetic feasibility. This scale and throughput is sufficient for prioritizing computationally enumerated analogue libraries from medicinal chemists or sets of novel compounds produced by generative artificial intelligence methodologies. Further acceleration of the search speed, potentially through computational hardware or modifications to the search algorithm, would be needed to efficiently evaluate compounds on a larger scale.

SynRoute was designed and built to focus on the type of drug-like compounds typically targeted across all phases of drug discovery programs. Where we have observed, limitations have been particularly around compounds with multiple chiral centers, such as complex natural products as well as less common ring systems that often require more specialized chemical transformations not well-covered by our templates. When tested against the four natural products described in the Chematica publication,^36^ complete routes were only found for one of the compounds, the natural product (−)dauricine. The other three targets returned only “partial routes” by SynRoute, meaning synthetic strategies were still displayed but not all intermediates could be fully traced back to purchasable feedstocks. In comparison, SynRoute was successfully able to find routes for seven out of eight targets described in the first Chematica paper on complex medically relevant targets.^9^ The only compound that did not return multiple routes was “BRD Inhibitor 8”, which contains a piperidin-2-one ring with two chiral centers that proved challenging. Examples of the top routes for all of the compounds are shown in the Supporting Information.

We have shown that SynRoute compared very favorably to two published benchmarks and used a much smaller set of transformations. This result shows that a good set of general enough transformations, combined with trained classifiers from the experimental literature, is likely the preferred approach for future retrosynthetic planning software tools.

## Data Availability

The data associated with this manuscript are in the manuscript and the Supporting Information.

## Supporting Information Available

The five 100-SMILES benchmarks are provided in the following five files: Benchmark1-smiles.csv: benchmark 1, 100 random SMILES from the ChEMBL database; Benchmark2-smiles.csv: benchmark 2, 100 random SMILES from the ChEMBL database; Benchmark3-smiles.csv: benchmark 3, 100 random SMILES from the ChEMBL database; Benchmark4-smiles.csv: benchmark 4, 100 random SMILES from the ChEMBL database; Benchmark5-smiles.csv: benchmark 5, 100 random SMILES from the ChEMBL database (ZIP)
Detailed computational benchmark results for the four expansion algorithms, detailed experimental schemes for the synthesis of itraconazole and bortezomib, screenshots of the graphical user interface of SynRoute, and examples of routes to complex medically relevant targets (PDF)
The name reactions currently implemented in SynRoute (TXT)
