# Supplementary material for: SynRoute: A Retrosynthetic Planning Software
Source: J Chem Inf Model. 2023 Aug 28;63(17):5484–95. doi: 10.1021/acs.jcim.3c00491 (PMC10498441; doi:10.1021/acs.jcim.3c00491)
Supplement: Supplementary file 2 — ci3c00491_si_002.pdf [file ci3c00491_si_002.pdf]

SynRoute™: a Retrosynthetic Planning Software

Mario Latendresse,* Jeremiah P. Malerich, James Herson, Markus Krummenacker, Judy Szeto, Vi-Anh Vu, Nathan Collins, and Peter B. Madrid

SRI International, 333 Ravenswood Ave, Menlo park, CA 94025, USA

E-mail: mario.latendresse@synfini.com,latendre@gmail.com

Supporting Information

Computational results

Table S1 presents the performance of four expansion algorithms on six 100-SMILES benchmarks extracted from the ChEMBL database using a maximum of 5K, 10K, 25K, and 50K generated reactions. An integer number in a cell indicates the number of SMILES, among 100, for which at least one route was found for one algorithm, one benchmark, and one maximum number of generated reactions. The best-first reaction algorithm is systematically the best performer across all the benchmarks for all maximum number of generated reactions.

Table S1: Performances of four expansion algorithms when generating reactions from templates.

Algorithm	AiZynth	Bench 1	Bench 2
Breadth-first	|49|57|62|63|	|50|60|66|67|	|65|68|70|71|
Depth-first	|54|61|67|71|	|47|61|66|67|	|70|72|79|81|
Best-first cpd	|61|64|68|72|	|57|61|65|69|	|68|68|72|76|
Best-first rxn	|**72**|**76**|**85**|**86**|	|**66**|**70**|**73**|**77**|	|**76**|**79**|**82**|**87**|
**Algorithm**	**Bench 3**	**Bench 4**	**Bench 5**
Breadth-first	|59|65|65|65|	|61|63|68|68|	|64|69|70|72|
Depth-first	|60|64|70|71|	|58|64|71|73|	|65|70|73|78|
Best-first cpd	|63|66|68|70|	|65|69|73|74|	|67|68|73|78|
Best-first rxn	|**71**|**73**|**77**|**78**|	|**69**|**72**|**79**|**79**|	|**76**|**79**|**85**|**90**|

Algorithm	Averages for 5K, 10K, 25K, 50K
Breadth-first	|58.0|63.6|66.8|67.6|
Depth-first	|59.0|65.3|71.0|73.5|
Best-first cpd	|63.5|66.0|69.8|73.1|
Best-first rxn	|**71.6**|**74.8**|**80.1**|**82.8**|

Experimental results

Itraconazole Synthesis

The itraconazole synthesis was done on the AutoSyn platform. Here we present some of the details of this synthesis.

Step 1) Alkylation to synthesize `itr-3` (see Figure S2).

Table S2: Run results of the first step to synthesize `itr-3`.

Amount recovered	286 mg
Production rate	343 mg / h
Yield	31%
Flow composition (NMR)	82% `itr-3`,<br>13% over-alkylation product
Time to product elution	1.5 h
Product collection window	50 min

A solution 2,4-dichlorophenacyl chloride `itr-1` (2.0 g, 9 mmol, 0.6 M) in NMP (15 mL) reacted with a solution of 1,2,4-triazole `itr-2` (3.1 g, 45 mmol, 3 M, 5 equiv.) in NMP

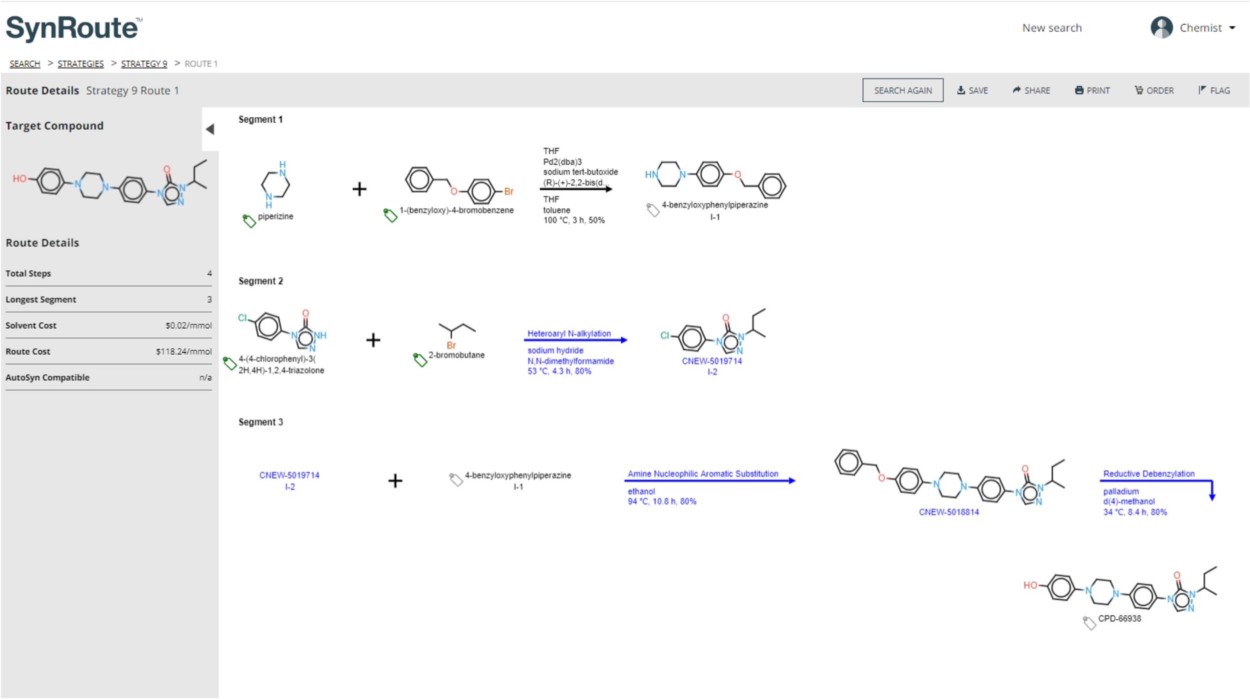

Figure S1: SynRoute route for synthesis of intraconazole triazolone intermediate.

(15 mL) at a total flow rate of 0.22 mL/min into a preheated Hastelloy reactor at 160 °C. Fractions containing `itr-3` were combined, concentrated to dryness, and purified by flash chromatography to afford `itr-3` (Figure S2 and Table S2). 1H-NMR(400 MHz, CDCl3) δ: 5.62 (s,2H); 7.38 (dd, J=4.0,8.0, 1H); 7.49 (d, J=4.0, 1H); 7.64 (d, J=8.0, 1H); 7.97 (s, 1H); 8.23 (s, 1H).

Step 2) Ketalization to synthesize `itr-7` (see Figure S3).

TfOH (1.4 mL, 16 mmol) was added to a solution of `itr-4` (1.08 g, 4.4 mmol, 1.1 equiv.) and triazolyl acetophenone `itr-3` (1 g, 3.9 mmol) in toluene (8 mL). Then the reaction mixture was stirred at room temperature for 60 h. The reaction was quenched by adding K_2CO_3 solution (5.2 g in 40.3 mL water) at 0 °C. The crude mixture was extracted with EtOAc (3×30 mL), washed with brine, and dried over Na_2SO_4. The solvent was removed and the residue was re-dissolved in EtOAc (2 mL). *p*-Toluenesulfonic acid monohydrate (834 mg, 4.4 mmol) in EtOAc (8 mL) was added dropwise to precipitate `itr-5` as a white solid (1.39 g, 74%) (Figure S3).

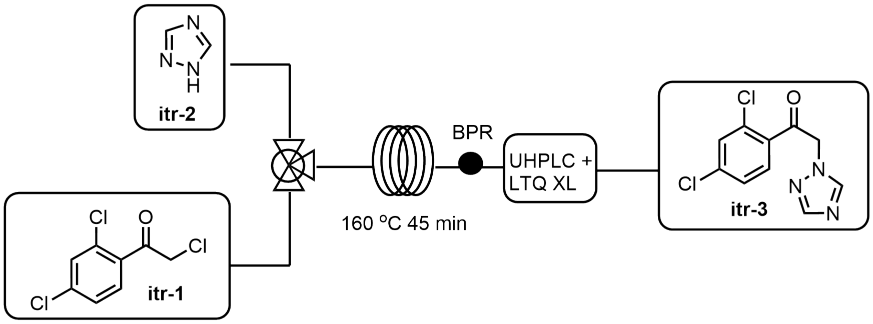

Figure S2: First step to synthesize `itr-3`.

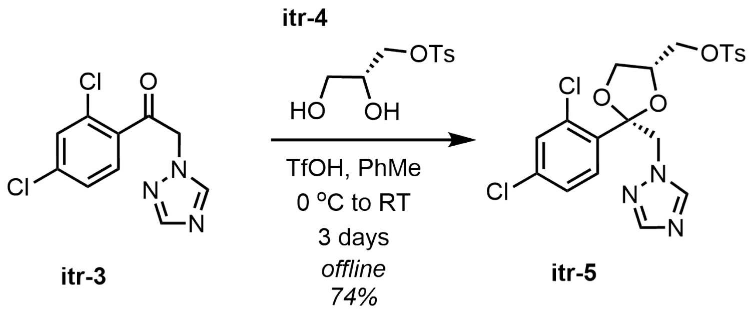

Figure S3: Ketalization to synthesize `itr-5`.

Step 3) Triazolidinone formation and alkylation to synthesize `itr-7` (Figure S4).

Table S3: Run results of triazolidinone formation and alkylation to synthesize `itr-7`.

Amount recovered	244 mg
Production rate	122 mg / h
Yield	30%
Flow composition (NMR)	52% itr-7, 27% itr-6,<br>21% unalkylated intermediate
Time to product elution	2.6 h
Product collection window	2 h

A solution of aniline `itr-6` (680 mg, 2.4 mmol, 0.3 M) and *p*TsOH (46 mg, 0.24 mmol, 10 mol%) in NMP (8 mL) was pumped into AutoSyn and met with a solution of methyl carbazate (1.5 g, 16.7 mmol, 2.1 M, 7.0 equiv.) and trimethyl orthoformate (0.92 mL, 8.4 mmol, 1.05 M, 3.5 equiv.) in NMP (7 mL) for a total flow rate of 0.166 mL/min. The combined streams were pumped through a preheated Hastelloy reactor (5 mL) at 140 °C. To the resulting mixture was added solution of NaOMe in MeOH (5.8 mL , 1.8 M, 6.0 equiv.)

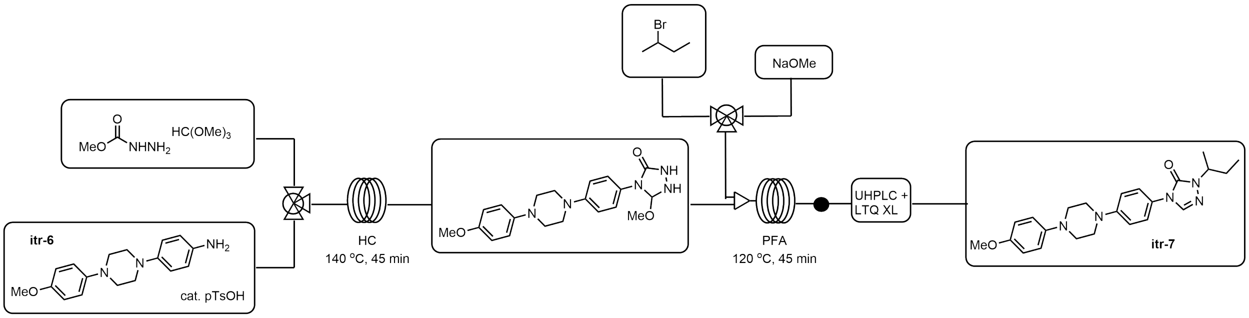

Figure S4: Triazolidinone formation and alkylation to synthesize `itr-7` on the AutoSyn platform.

in MeOH (9.2 mL) and a solution of 2-bromobutane (3 mL, 8.4 mmol, 6.0 equiv.) in NMP (12 mL) at total flow rate of 0.32 mL/min. The combined mixture was pumped through a preheated PFA reactor (10 mL) at 120 °C. Fractions of effluent from AutoSyn containing `itr-7` were concentrated to dryness and purified by flash chromatography (Figure S4 and Table S3). 1H-NMR(400 MHz, d6DMSO) δ: 0.73 (t, J=6.8, 3H); 1.01 (m, 1H); 1.24 (d, J=6.8, 2H); 3.09 (m, 4H); 3.25 (m, 4H); 3.64 (s, 3H); 6.46 (d, J=8.0, 2H); 6.68-6.85 (m, 4H); 7.0 (d, J=7.6, 2H); 7.42 (d, J=7.6, 2H); 8.26 (s, 1H).

Step 4) Demethylation to synthesize `itr-8` (see Figure S5).)

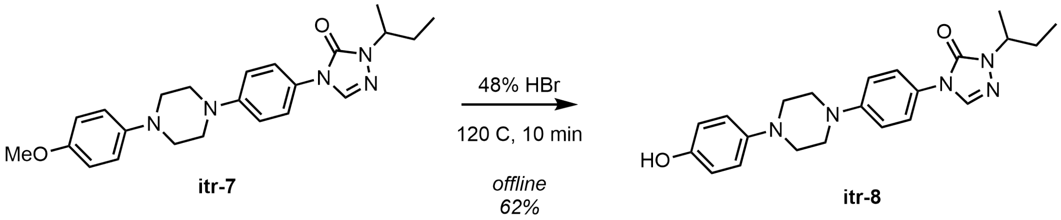

Figure S5: Demethylation to synthesize `itr-8` on the AutoSyn platform.

Compound `itr-7` (244 mg, 0.6 mmol) was suspended in 48% HBr (25 mL) and was heated to 120 °C for 12 h. After the reaction was completed, the mixture was cooled and neutralized with 1 M NaOH under 0 °C. It was extracted with chloroform (3 X 10 mL). The organic layer was washed with water (3 X 15 mL), sat. aqueous NaCl (2 x 15 mL), and dried with Na_2SO_4. The crude was purified by flash chromatography to give `itr-8` (151 mg, 62%)

(see Figure S5).

Step 5) *O*-Alkylation to synthesize itraconazole (see Figure S6).

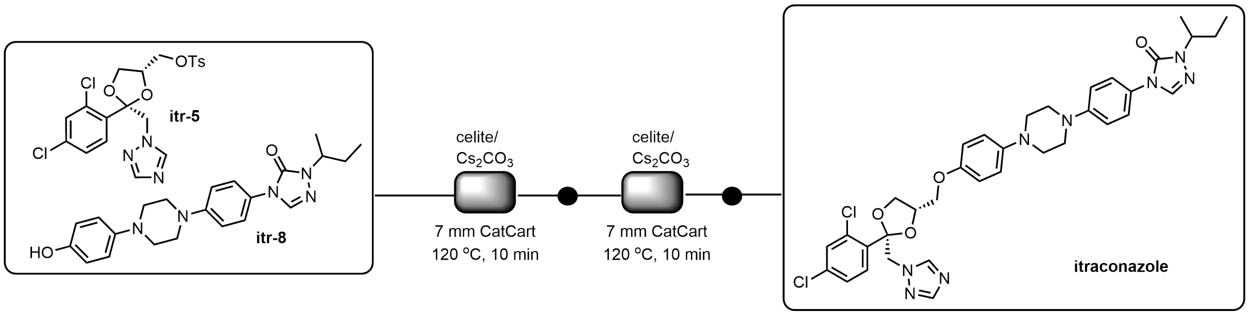

Figure S6: Last step to synthesize itraconazole on the AutoSyn platform.

Table S4: Run results of step 5 to synthesize itraconazole.

Amount recovered	50 mg
Production rate	50 mg / h
Yield	30%
Flow composition (NMR)	60% itraconazole, 40% starting materials
Time to product elution	2.6 h
Product collection window	2 h

A solution of `itr-5` (0.04 M) and `itr-8` (0.04 M) in DMSO was pumped into AutoSyn and through two successive packed bed reactors containing 9:1 Cs_2CO_3:Celite. Fractions of effluent from AutoSyn containing itraconazole were diluted with brine (50 mL) and extracted with CH_2Cl_2 (3x50 mL). The combined organic extracts were dried over Na_2SO_4, filtered, and concentrated. The residue was purified by flash chromatography to give itraconazole (see Figure S6 and Table S4).

Table S5: Detailed comparison of SynRoute recommended reaction conditions and the conditions used in the laboratory to synthesize itraconazole via the AutoSyn automated flow chemistry platform.

**Product formed in reaction**	**Reaction Data Source**	**SynRoute Recommended Conditions**	**Experimental Conditions**	**Rational for Modfications**
itr-3	US9095589	itr-1 (1M, 1 eq.)<br>itr-2 (2M, 2 eq.)<br>NaHCO3 (1M, 1 eq.)<br>toluene<br>100C, 3 h<br>53% yield	*Performed on AutoSyn flow chemistry platform*<br>itr-1 (0.6M, 1 eq.)<br>itr-2 (3M, 5 eq.)<br>NMP<br>160C, 0.75 h<br>31% yield	Increase imidazole equivalents to eliminate need for solid base.<br>Higher heat and shorter reaction time for higher production rate.
itr-5	US9095589	itr-3 (0.4M, 1 eq.)<br>(S)-1-tosyloxy-2,3-propanediol (0.4M, 1 eq.)<br>TfOH (1.6M, 4.1 eq.)<br>toluene<br>rt, 60 h<br>55% yield	itr-3 (0.5M, 1 eq.)<br>(S)-1-tosyloxy-2,3-propanediol (0.5M, 1 eq.)<br>TfOH (2M, 4.1 eq.)<br>toluene<br>rt, 60 h<br>74% yield	Identical conditions to literature
itr-7	SynRoute Computer Generated Reaction "Heteroaryl N-alkylation"	triazolone (1 eq.)<br>2-bromobutate (1 eq.)<br>NaH (1 eq.)<br>DMF<br>53C, 4.3h<br>80%	*Performed on AutoSyn flow chemistry platform*<br>triazolone (1 eq.)<br>2-bromobutate (6 eq.)<br>NaOMe in Methanol (1.8M, 6 eq.)<br>NMP<br>120C, 45 min	Switched base from NaH to NaOMe for homogeneity.<br>Increased 2-bromobutate equivalents for faster production rate.
itr-8	Different protecting group			Switched to methoxy protecting group rather than benzyl
itraconazole	US9095589	itr-5 (0.21M, 1.1 eq.)<br>itr-8 (0.2M, 1 eq.)<br>KOH (0.78M, 3.9 eq.)<br>DMF<br>50C, overnightHi<br>45%	*Performed on AutoSyn flow chemistry platform*<br>itr-5 (0.04M, 1 eq.)<br>itr-8 (0.04M, 1 eq.)<br>Cs2CO3 (packed bed cartridge)<br>DMSO<br>30% yield	Decreased concentrations for greater reactant solubility.R

Bortezomib Synthesis

The bortezomib synthesis was done on the AutoSyn platform. We present some of the details of this synthesis.

Steps 1 and 2) 1st amide coupling and deprotection on the AutoSyn platform to synthesize dipeptide carboxylic acid `bor-2` (see Figure S7).

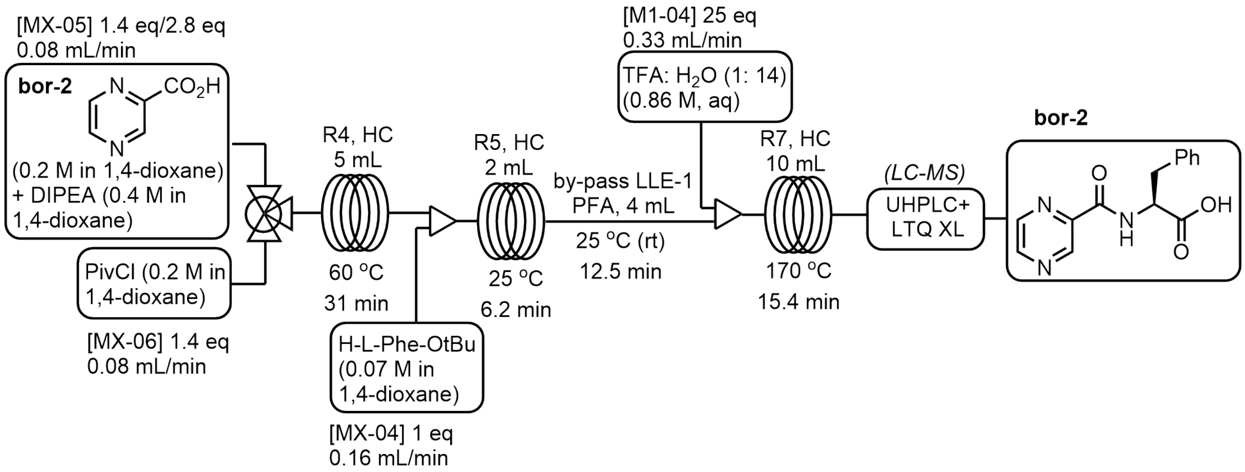

Figure S7: 1st amide coupling and deprotection on the AutoSyn platform to synthesize dipeptide carboxylic acid `bor-2`.

Table S6: Run results of 1st amide coupling and deprotection to synthesize dipeptide carboxylic acid `bor-2`.

Amount recovered	324 mg
Production rate	177 mg / h
Yield	97%
Purity (NMR)	97%
Time to product elution	2 h
Product collection window	110 min

Pump MX-05 was primed with a solution of 2-pyrazinecarboxylic acid (0.2 M in 1,4-dioxane) and DIPEA (0.4 M in 1,4-dioxane), and pump MX-06 was primed with a solution of pivaloyl chloride (0.2 M in 1,4-dioxane). These solutions were connected via a T-mixer, pumped into inlet 5 at 0.08 mL/min each into a Hastelloy reactor (R4, 5 mL, 31 min residence time) heated at 60 °C. After 20 minutes, pump MX-04 primed and delivered

L-phenylalanine *tert*-butyl ester (0.07 M in 1,4-dioxane) to the system at inlet 6 at 0.16 mL/min. L-Phenylalanine *tert*-butyl ester met the flow of pivalic pyrazine-2-carboxylic anhydride before entering a Hastelloy reactor (R5, 2 mL) and PFA reactor (4 mL, between V11 and V18) at room temperature for a total of 18 min residence time. Pump M1-04, primed with TFA (0.86 M, aqueous), began flowing 47 min after initiation of pump MX-05 (2-pyrazinecarboxylic acid). TFA was introduced (0.33 mL/min) into the system at inlet 10 and met with dipeptide *tert*-butyl ester before entering a Hastelloy reactor (R7, 10 mL, 15.4 min residence time) heated to 170 °C. Reactor effluent was directed to LC-MS for online analysis. Fractions containing pyrazinylcarbonyl-L-phenylalanine were collected over 110 min and subjected to aqueous work-up. Extracted with EtOAc, washed with water (2x) and brine. The organic layer was dried with sodium sulfate, filtered, and concentrated to afford dipeptide carboxylic acid `bor-2` (324 mg, 97% yield). 1H-NMR(400 MHz, CDCl3) δ: 3.78 (s, 2H); 5.07 (m, 1H); 7.1-7.3 (m, 5H); 8.28 (d, J=8.0, 1H); 8.69 (d, J=2.7, 1H); 8.79 (d, J=2.7, 1H); 9.31 (s, 1H).

Step 3) 2nd amide coupling step on the AutoSyn platform to synthesize bortezomib pinanediol `bor-4` (see Figure S8).

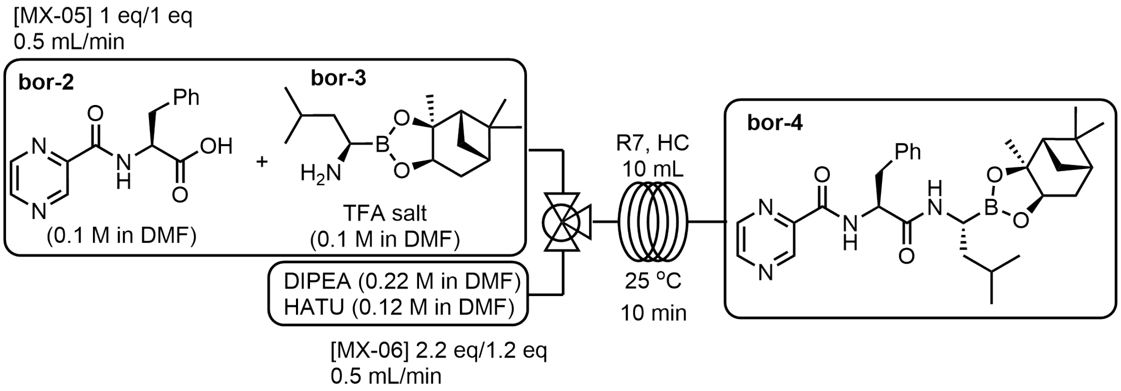

Figure S8: 2nd amide coupling reaction on the AutoSyn platform to synthesize bortezomib pinanediol `bor-4`.

Pump MX-05 was primed with a solution of pyrazinylcarbonyl-L-phenylalanine (0.1 M in DMF) and (*R*)-boroleucine-(1*S*,2*S*,3*R*,5*S*)-(+)-pinanediol ester trifluoroacetate (0.1 M in

Table S7: Run results of 2nd amide coupling reaction to synthesize bortezomib pinanediol `bor-4`.

Amount recovered	324 mg
Production rate	486 mg / h
Yield	51%
Purity (NMR)	100%
Time to product elution	20 min
Product collection window	40 min

DMF) and pump MX-06 was primed with a solution of HATU (0.12 M in DMF) and DIPEA (0.22 M in DMF). These solutions were connected via a T-mixer, pumped into inlet 10 at 0.5 mL/min each into a Hastelloy reactor (R7, 10 mL, 10 min residence time) at room temperature. Fractions containing bortezomib pinanediol `bor-4` were collected over 40 min and subjected to aqueous workup. All fractions were combined and citric acid was added (10%, aq) with EtOAc (3x). Combined organic layer and washed with water (2x) and brine. The organic layer was dried with sodium sulfate. Filtration and solvent evaporation gave red-orange oil, which was chromatographed with 40% EtOAc in hexanes to obtain bortezomib pinanediol `bor-4` (345 mg, 51% yield). 1H-NMR(400 MHz, CDCl3) δ: 0.81 (t, J=8.0, 9H); 1.25 (m, 6H); 1.36 (m, 4H); 1.89 (m, 2H); 2.04 (s, 3H); 2.16 (m, 1H); 2.31 (m, 1H); 3.15 (m, 6H); 4.12 (q, J=8.0, 2H); 4.29 (d, J=8.0, 1H); 4.80 (q, J=8.0, 1H); 5.91 (d, J=8.0, 1H);7.1-7.3 (m, 5H); 8.34 (d, J=8.0, 1H); 8.52 (d, J=2.4, 1H); 8.74 (d, J=2.4, 1H); 9.34 (s, 1H).

Step 4) 2nd deprotection on the AutoSyn platform to synthesize bortezomib (see Figure S9).

Table S8: Run results of 2nd deprotection to synthesize bortezomib.

Amount recovered	135 mg
Production rate	68 mg / h
Yield	70%
Purity (NMR)	90%
Time to product elution	60 min
Product collection window	80 min

Pump MX-01 was primed with a solution of *iso*butylboronic acid (0.4 M in 1,4-dioxane), pump MX-02 was primed with a solution of HCl (0.3 M, aq), and pump MX-06 was primed

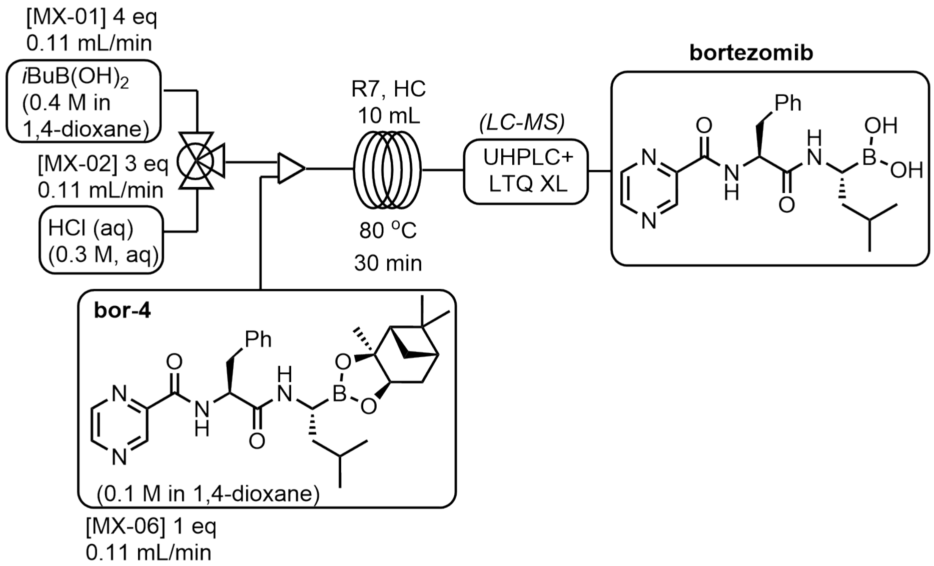

Figure S9: 2nd deprotection on the AutoSyn platform to synthesize bortezomib.

with bortezomib pinanediol `bor-4` (0.1 M in 1,4-dioxane). These solutions were connected via a T-mixer and pumped into inlet 10 at 0.11 mL/min each into a Hastelloy reactor (R7, 10 mL, 30 min residence time) at 80 °C. Reactor effluent was directed to LC-MS for online analysis. Fractions containing bortezomib were collected over 80 min and subjected to aqueous workup. The combined fractions were washed with hexanes (2x). The bottom layer contained bortezomib in 1,4-dioxane was concentrate to dryness. Then the reaction mixture was extracted with DCM, washed with $NaHCO_3$ (sat'd, aq) and brine. The organic layer was dried with sodium sulfate. Filtration and solvent evaporation gave bortezomib (135 mg, 70% yield).

Template examples implemented in SynRoute

The Table S9 lists some examples of reaction names and their templates, as SMARTS, implemented in SynRoute to generate reactions retrosynthetically. These name reactions are part of the 263 name reactions implemented in SynRoute. Some reaction names require several SMARTS. These SMARTS have been designed to work in the forward and backward (retrosynthetically) directions.

Table S9: Examples of templates implemented in SynRoute

Reaction Name	Templates as one or more SMARTS
Nitro reduction to amine	[C,c:1][N+:2](=O)[O-]>>[C,c:1][NH2+0;D1:2]
Nitrile to acid hydrolysis	[C,c:1][C:2]#N>>[C,c:1][C:2](=O)[OH]
Boronic acid formation	[CX3,cX3:1]Br>>[CX3,cX3:1]B([OH])[OH]
Ketone or aldehyde reduction	[C,c:3][CX3H1:1](=[O:2])>>[C,c:3][CX4H2:1]([OH:2])
	[C,c:3][CX3:1](=[O:2])[C,c:4]>>[C,c:3][CX4H:1]([OH:2])[C,c:4]
Electrophilic Aromatic nitration	[ch:1]1[c,n:2][c,n:3][c,n:4][c,n:5]1>> [NX3+](=O)([O-])[c:1]1[c,n:2][c,n:3][c,n:4][c,n:5]1
	[ch:1]1[c,n:2][c,n:3][c,n:4][c,n:5][c,n:6]1>> [NX3+](=O)([O-])[c:1]1[c,n:2][c,n:3][c,n:4][c,n:5][c,n:6]1
Imine reduction	[*:2][N:1]=[C:3]([*:4])[*:5]>>[*:2][NH:1][CH:3]([*:4])[*:5]
Epoxidation	[C,c:3][C:1]=[C:2]>>[C,c:3][C:1]1[C:2]O1
Ester reduction to alcohol	[C:4][#6:2](=[O:1])[O:3][C]>>[C:4][#6H2:2]([OH:1])
	[C:4][#6:2](=[O:1])[OH:3]>>[C:4][#6H2:2]([OH:1])
Ketone epoxidation	[C:1]([C,c:3])([C,c:4])=[O:2]>> [C:1]1([C,c:3])([C,c:4])[O:2][CH2]1
Nitrile to imine	[C,c:3][C:2]#[N:1]>>[C,c:3][CH:2]=[NH:1]
Alkyne to alkane	[C,c:3][C:1]#;!@[C:2][C,c:4]>>[C,c:3][CH2:1]-;!@[CH2:2][C,c:4]
Alcohol to acid	[C,c:3][CH2:1][OH:2]>>[C,c:3][C:1](=O)[OH:2]
Acid to acid chloride	[C,c:1][C:2](=[O:3])[OH:4]>>[C,c:1][C:2](=[O:3])[Cl:4]
Amide to imidoyl chloride	[#6;$([#6]-[#6,#1]):1](=O)[#7;$([#7]-[#6,#1]):2]>> [#6:1](Cl)=[#7:2]
Allylic oxidation	[Ch;X4:1][CX3:2]=[CX3:3]>>[OH][CX4:1][CX3:2]=[CX3:3]

Screenshots of SynRoute's Graphical User Interface

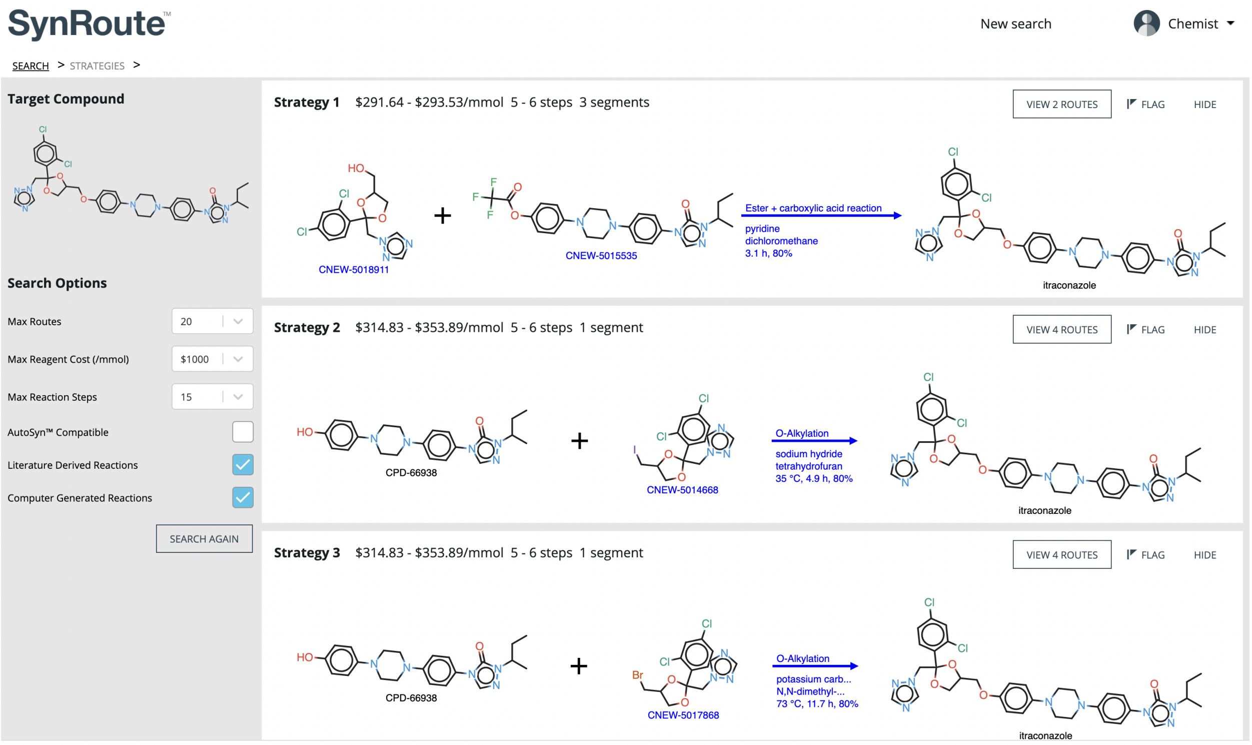

Figure S10: The display of strategies on the *Strategies Page* following a search to synthesize a target compound.

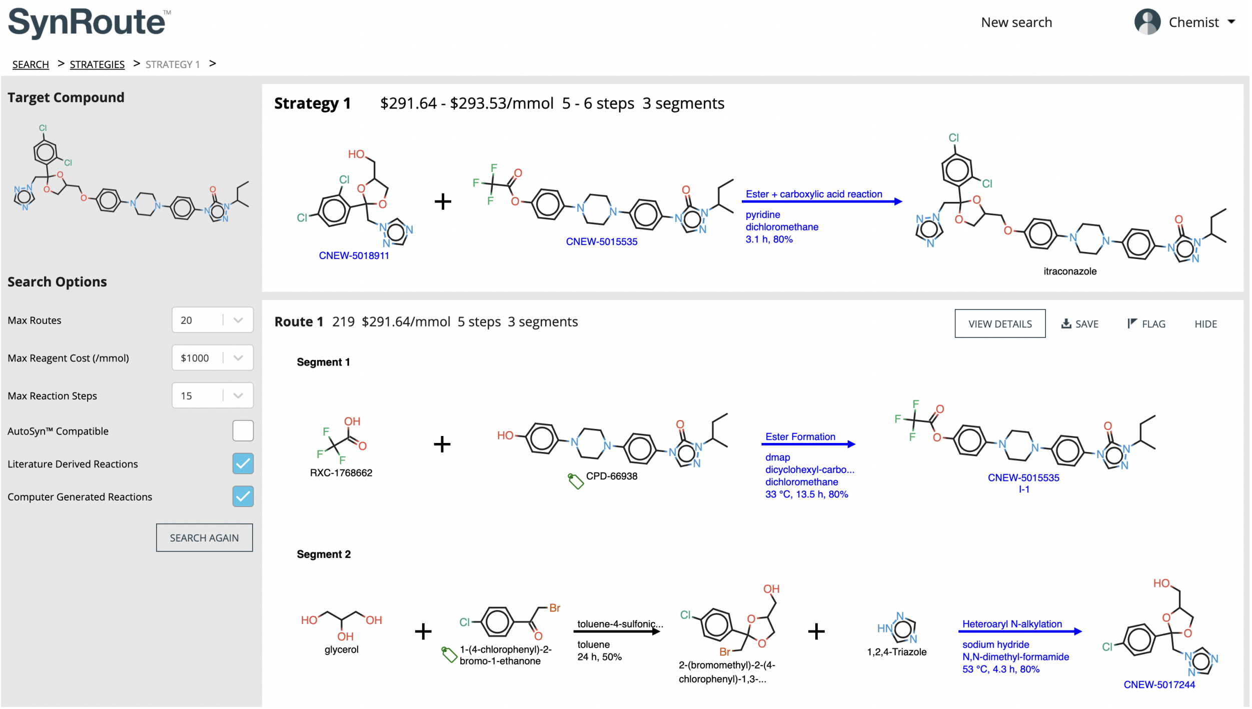

Figure S11: The display on the *Strategy Page* showing one strategy selected.

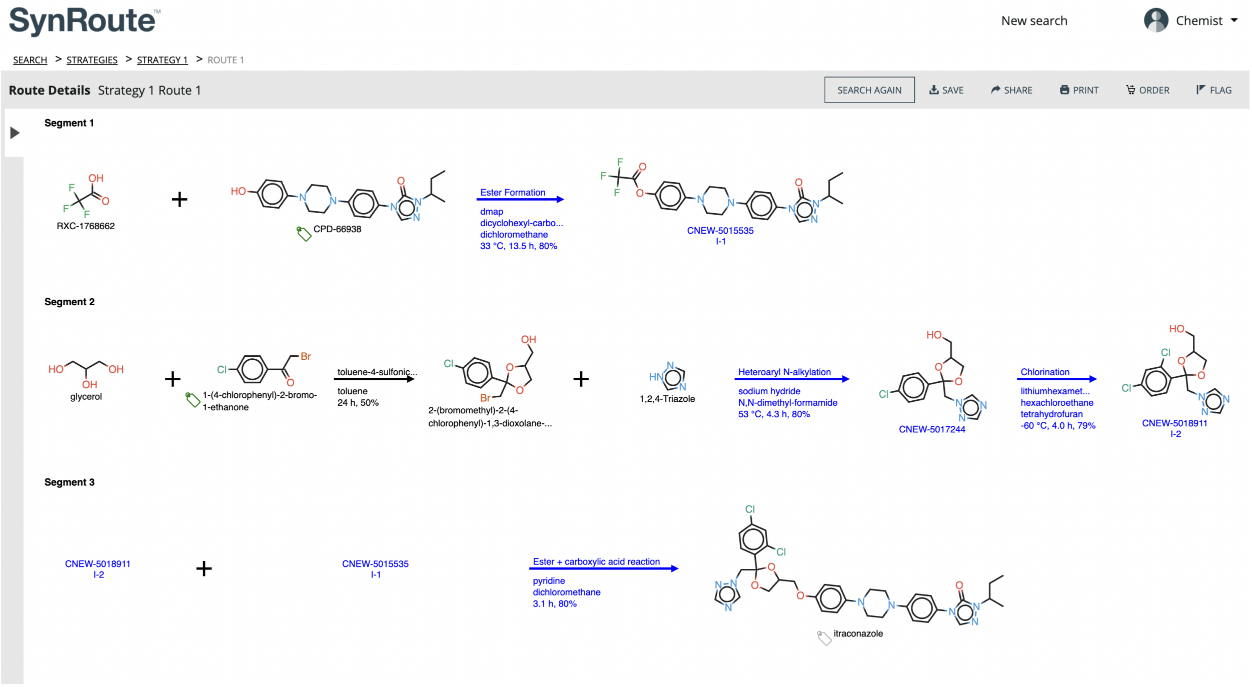

Figure S12: The display on the *Route Page* of one route selected from the *Strategy Page*.

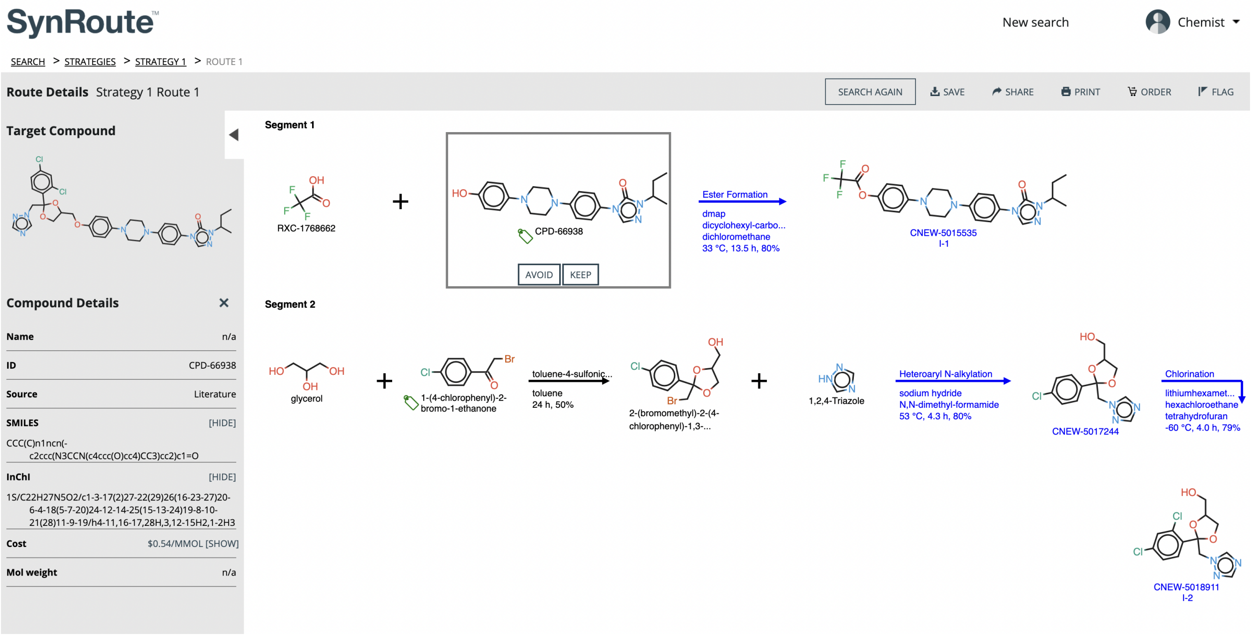

Figure S13: The display on the left-side panel shows data about a compound selected from the *Route Page*.

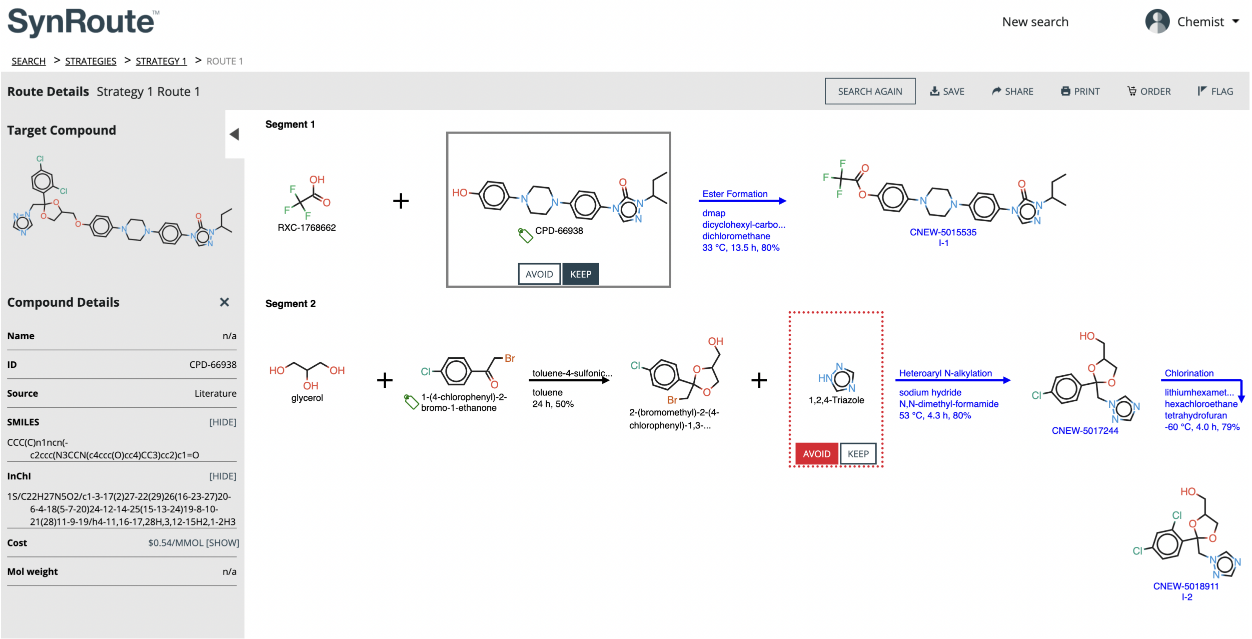

Figure S14: The buttons *keep* and *avoid* selected on two compounds on the *Route Page*.

Examples of SynRoute Routes to Complex Medically-Relevant Targets

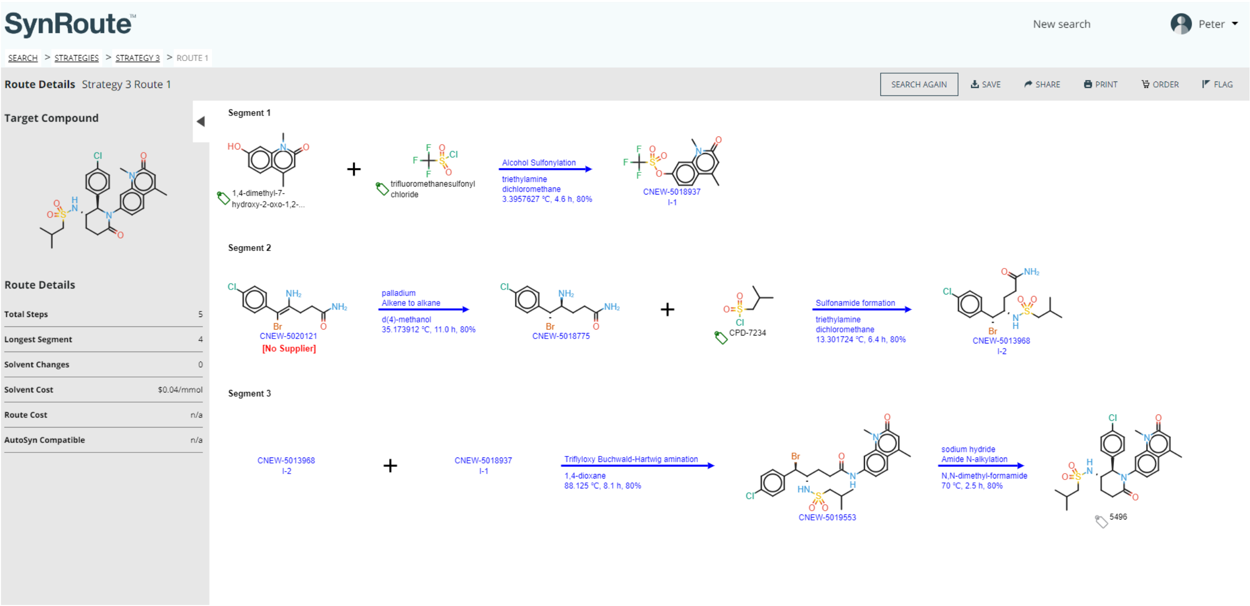

Figure S15: Example of a top route for a BRD 7/9 inhibitor.

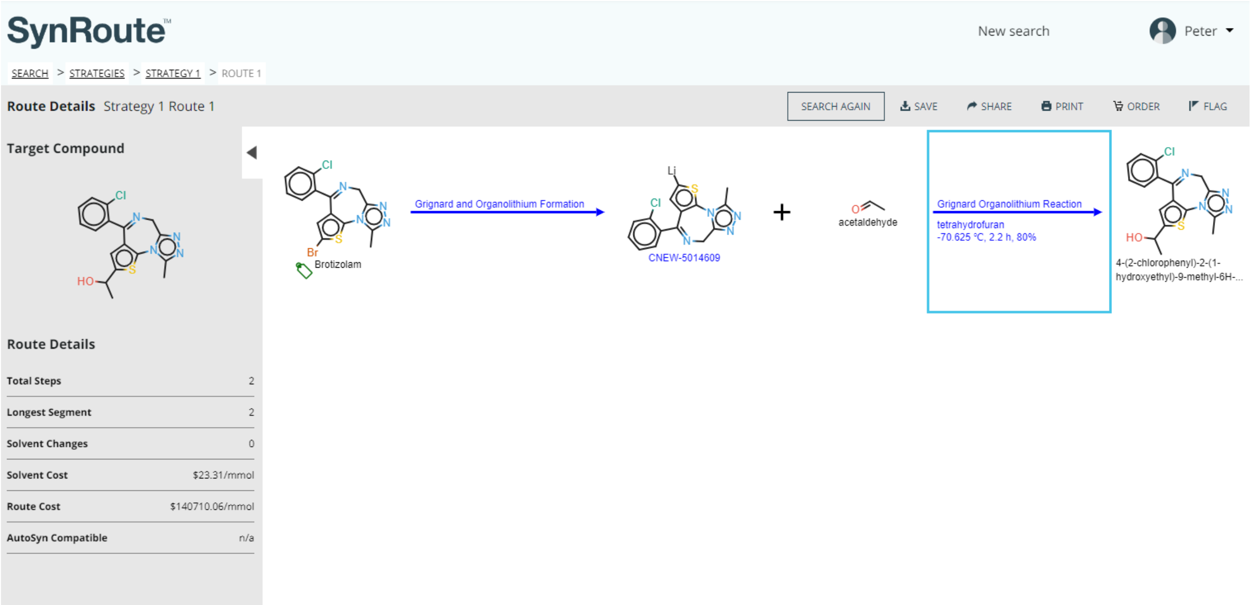

Figure S16: Example of a top route for alpha-hydroxyetizolam.

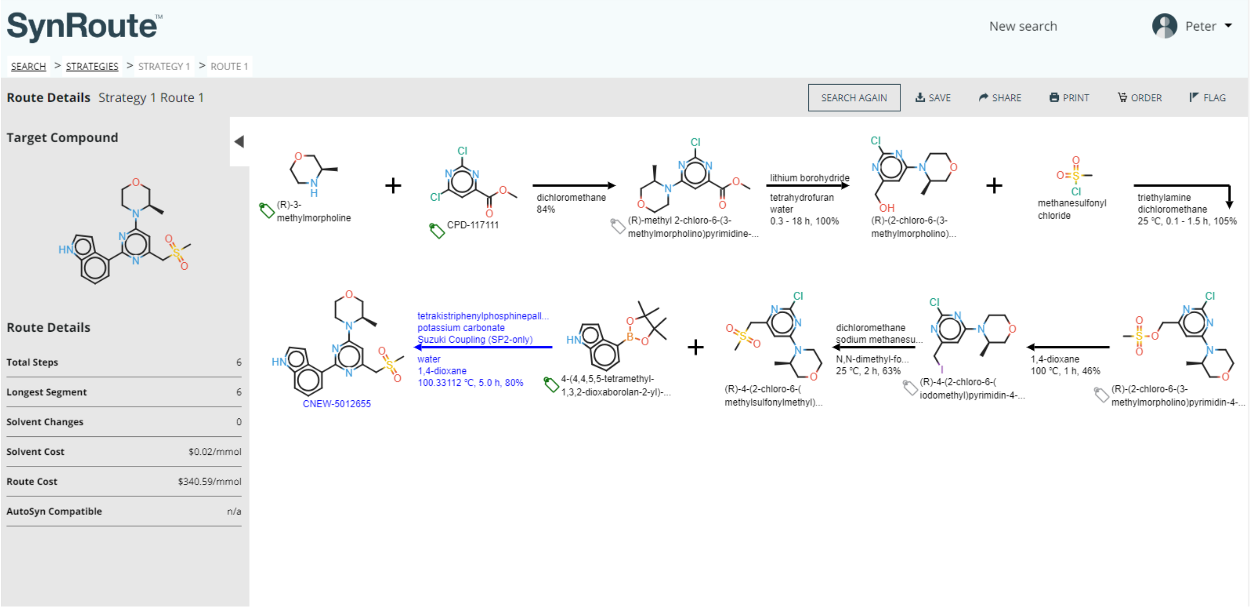

Figure S17: Example of a top route for an ATR kinase inhibitor.

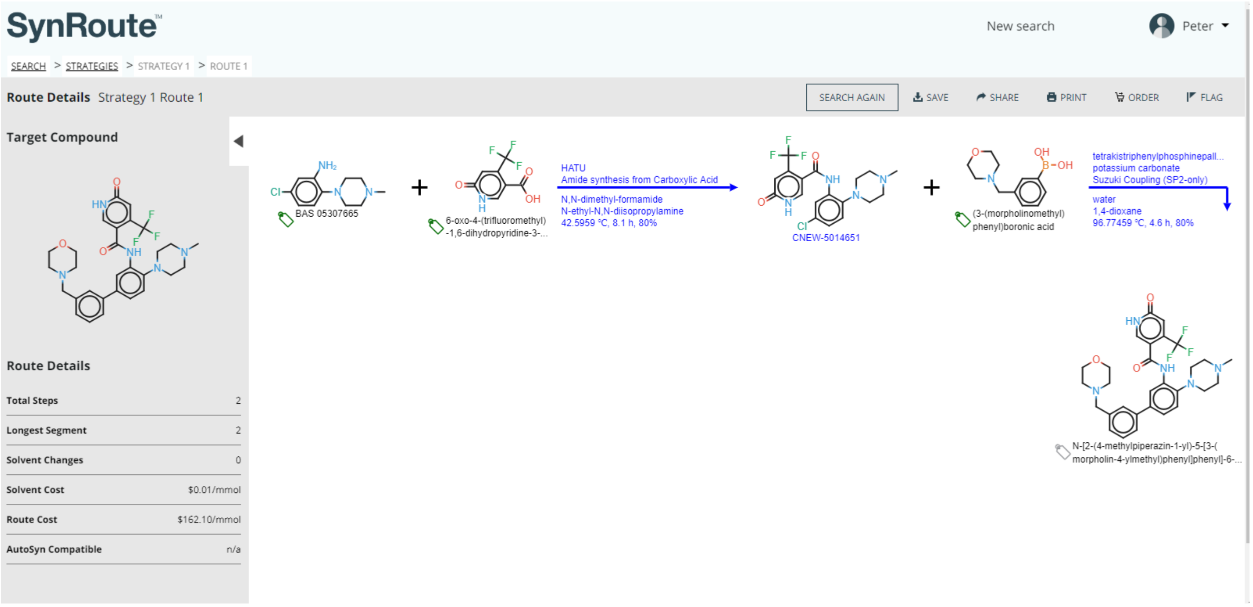

Figure S18: Example of a top route for a human acute-myloid-leukemia inhibitor.

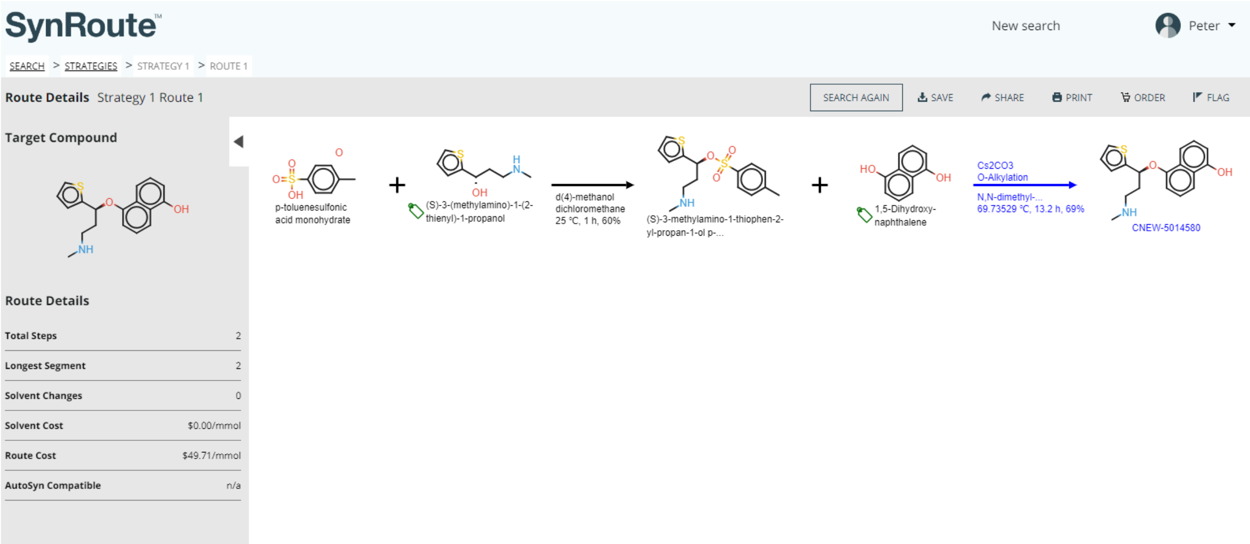

Figure S19: Example of a top route for (S)-4-hydroxyduloxetine.

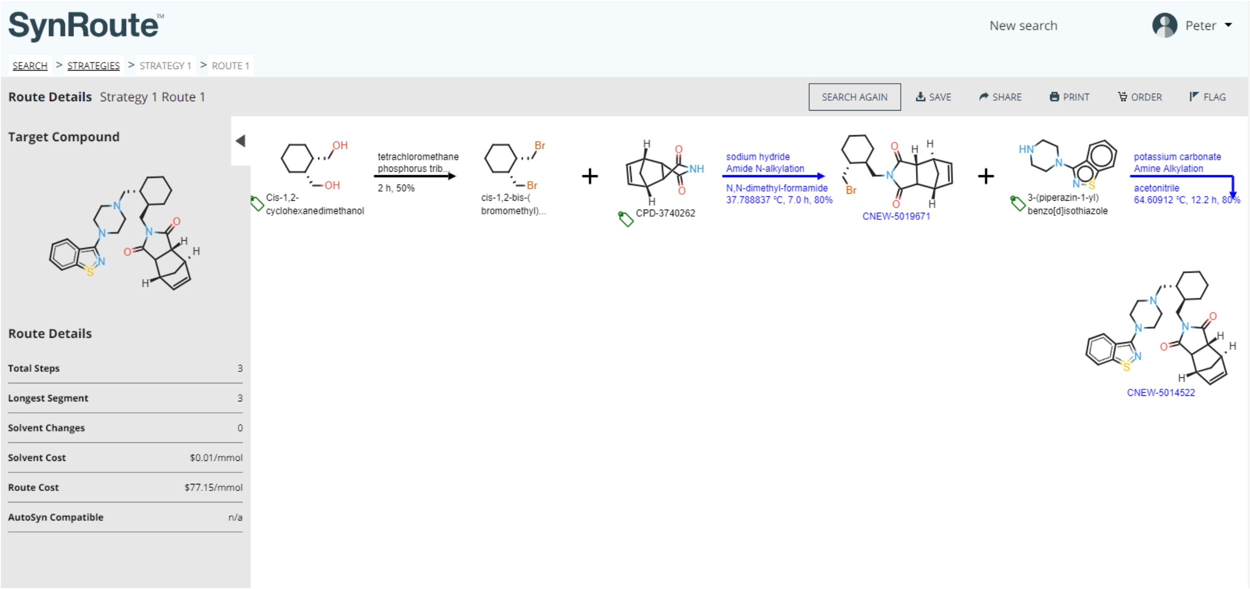

Figure S20: Example of a top route for 5-beta/6-beta-hydroxyurasidone.

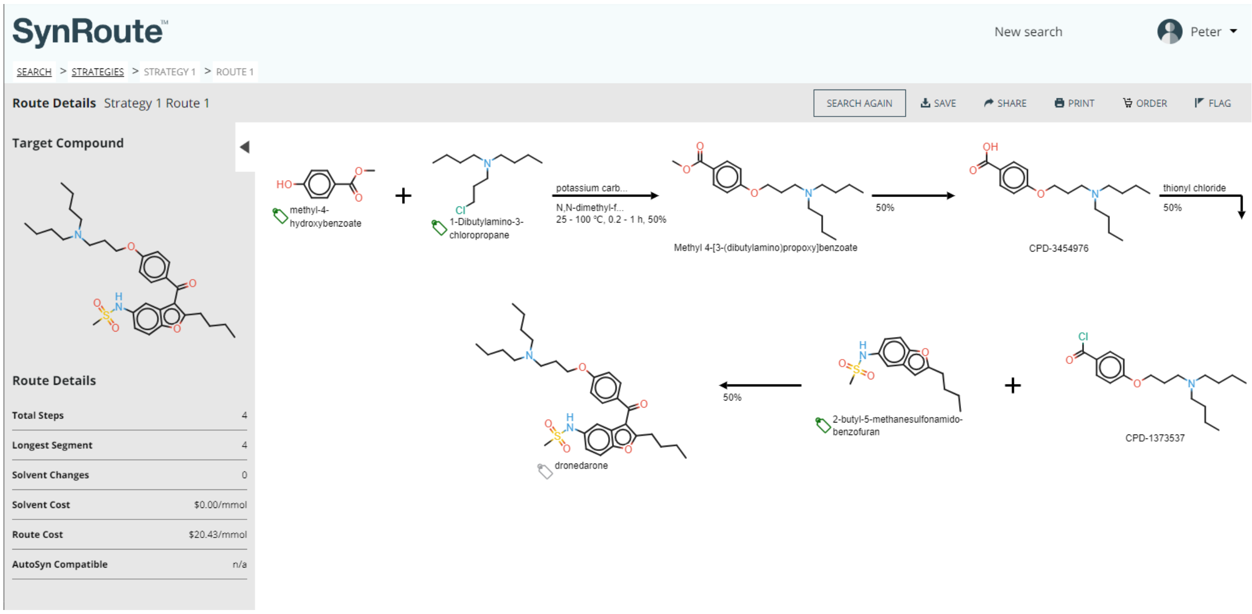

Figure S21: Example of a top route for dronedarone.

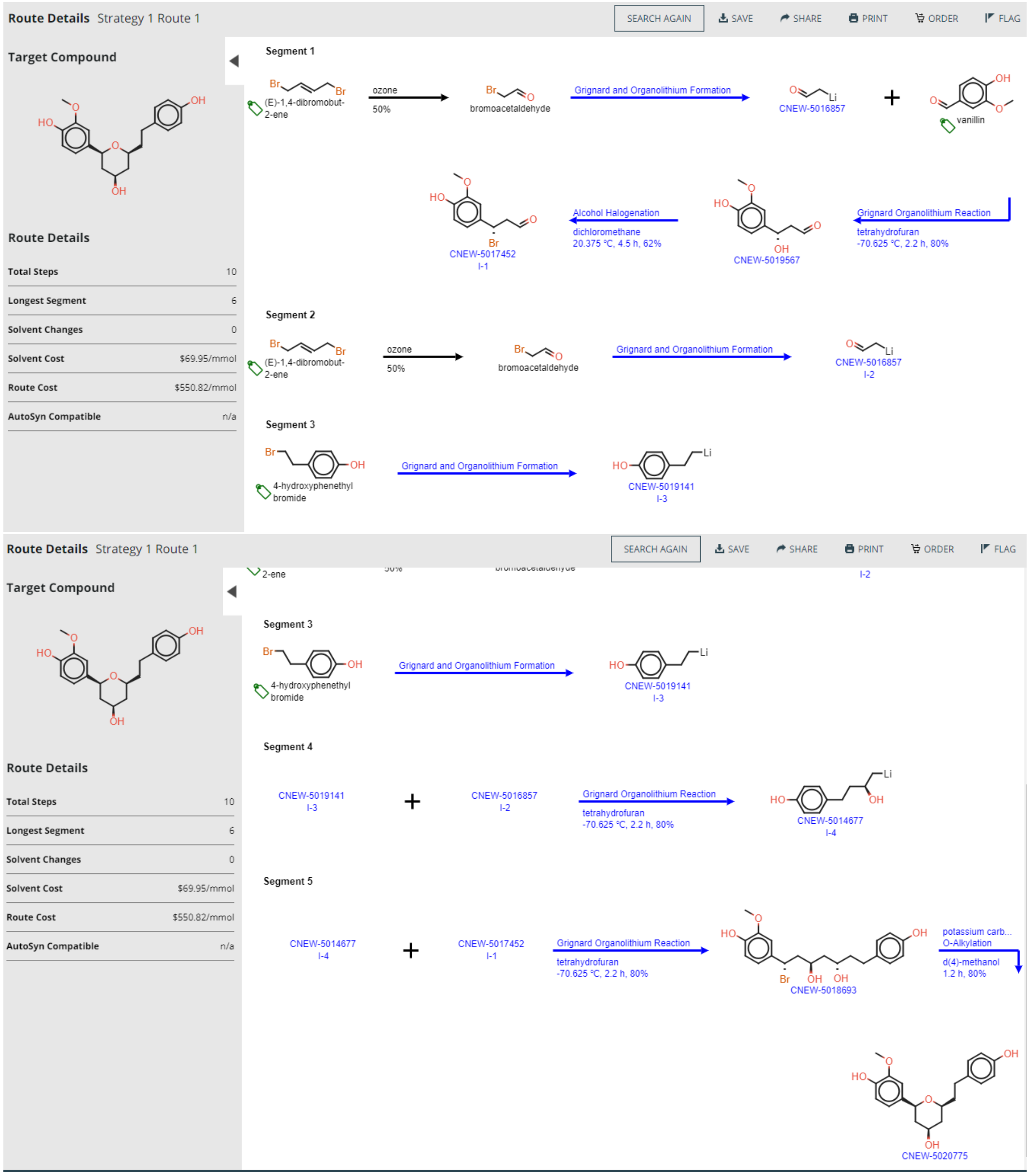

Figure S22: Example of a top route for engelheptanoxide.

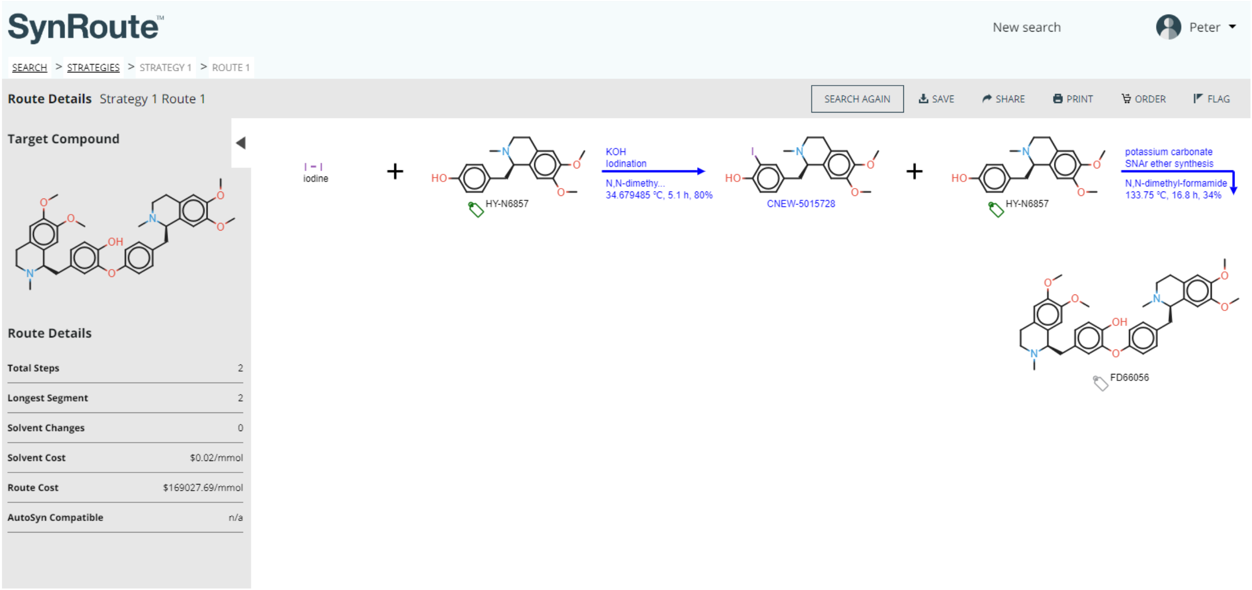

Figure S23: Example of a top route for the synthesis of the natural product dauricine.
